# Characterising the Transcriptional and Translational Impact of the Schizophrenia-Associated miR-1271-5p in Neuronal Cells

**DOI:** 10.3390/cells9041014

**Published:** 2020-04-18

**Authors:** Dylan J. Kiltschewskij, Michael P. Geaghan, Murray J. Cairns

**Affiliations:** 1 School of Biomedical Sciences and Pharmacy, University of Newcastle, Callaghan 2308, Australia; dylan.kiltschewskij@uon.edu.au (D.J.K.); michael.geaghan@newcastle.edu.au (M.P.G.); 2 Centre for Brain and Mental Health Research, Hunter Medical Research Institute, New Lambton 2305, Australia; 3 Schizophrenia Research Institute, Randwick 2031, Australia

**Keywords:** microRNA, mRNA sequencing, ribosome profiling, translation, neuron

## Abstract

MicroRNA (miRNA) coordinate complex gene expression networks in cells that are vital to support highly specialised morphology and cytoarchitecture. Neurons express a rich array of miRNA, including many that are specific or enriched, which have important functions in this context and implications for neurological conditions. While the neurological function of a number of brain-derived miRNAs have been examined thoroughly, the mechanistic basis of many remain obscure. In this case, we investigated the transcriptome-wide impact of schizophrenia-associated miR-1271-5p in response to bidirectional modulation. Alteration of miR-1271-5p induced considerable changes to mRNA abundance and translation, which spanned a diverse range of cellular functions, including directly targeted genes strongly associated with cytoskeletal dynamics and cellular junctions. Mechanistic analyses additionally revealed that upregulation of miR-1271-5p predominantly repressed mRNAs through destabilisation, wherein 3′UTR and coding sequence binding sites exhibited similar efficacy. Knockdown, however, produced no discernible trend in target gene expression and strikingly resulted in increased expression of the highly conserved miR-96-5p, which shares an identical seed region with miR-1271-5p, suggesting the presence of feedback mechanisms that sense disruptions to miRNA levels. These findings indicate that, while bidirectional regulation of miR-1271-5p results in substantial remodeling of the neuronal transcriptome, these effects are not inverse in nature. In addition, we provide further support for the idea that destabilisation of mRNA is the predominant mechanism by which miRNAs regulate complementary mRNAs.

## 1. Introduction

MicroRNAs (miRNAs) are a class of small, non-coding RNAs, which coordinate gene expression networks via post-transcriptional repression of mRNAs [1]. Tissue profiling studies have established that these molecules are considerably enriched within neurons, wherein they display complex patterns of spatial and temporal distribution [2,3,4,5,6]. miRNA have substantial regulatory capacity and have been shown to be vital for coordinating a number of neural processes from the timing of neurogenesis and migration through the regulation of neurotransmission and synaptic plasticity [7,8,9,10]. Dysregulation of individual miRNAs can, therefore, result in changes to neuronal structure and function [11], and, given that miRNAs can directly target thousands of genes, their effects can be quite profound [12]. Recent evidence has revealed that disturbances to miRNA function in the neuronal context are a consistent feature in psychiatric disorders, such as schizophrenia, major depression, and autism spectrum disorders, which suggests a potentially important role in pathogenesis or symptomology [13,14]. Characterising gene expression networks regulated by affected miRNAs is, therefore, crucial for understanding their functional contribution to neurobiological processes and disease. While the number of profiled neuronal miRNAs is continually expanding, the vast majority remain largely uncharacterised.

One miRNA with considerable functional potential in neurons is miR-1271-5p, which is a paralog of the highly conserved and extensively described miR-96-5p [15]. Previous low-throughput analysis of miR-1271-5p has shown this miRNA exhibits expression in the human forebrain and additionally regulates the expression of genes involved in neuronal signaling, which indicates a potentially important role in neuronal physiology [15]. These findings have been extended through recent high-throughput sequencing studies from our group in which miR-1271-5p has been found to be significantly downregulated in the peripheral blood mononucleocytes (PBMCs) of individuals with schizophrenia [16]. This process replicates previous results from a microarray study [17]. In addition, we also detected upregulation of the miRNA after K^+^ depolarisation of neuronally differentiated SH-SY5Y cultures (Kiltschewskij et al., submitted). This evidence suggests that miR-1271-5p may contribute to neuronal physiology. However, additional evidence pertaining the effects of this miRNA on neuronal gene expression is lacking. Therefore, to gain further insight into the gene expression networks regulated by this miRNA and determine the mechanistic basis of target mRNA regulation, we explored patterns of mRNA expression and translation in response to bi-directional modulation of miR-1271-5p in vitro and observed significant changes associated with regulation of cytoskeletal dynamics and cellular junctions. In addition, our results show that, while miR-1271-5p overexpression predominantly results in repression of target mRNAs via degradation, knockdown produces a comparatively complex disruption of the post-transcriptional landscape and strikingly impacts the expression of miRNAs with similar seed sequences.

## 2. Materials and Methods

### 2.1. Cell Culture

SH-SY5Y human neuroblastoma cells were grown and maintained in Dulbecco’s Modified Eagle’s Medium (DMEM, HyClone, Logan, UT, USA) supplemented with 10% heat-inactivated foetal bovine serum (Bovogen Biologicals, Essendon, VIC, Australia), 2% HEPES (Gibco, Carlsbad, CA, USA), and 1% L-glutamine (Invitrogen, Carlsbad, CA, USA). Cultures were maintained at 37 °C in a 95% oxygen, 5% carbon dioxide, 90% humidity atmosphere and passaged regularly by washing with phosphate buffered saline (PBS, Gibco) and trypsinisation (0.1% trypsin-EDTA in PBS, Gibco). All cells used in this study were passage 11 at the time of harvest. All experiments were conducted in biological quadruplicate with the exception of ribosome profiling, which was performed in triplicate.

### 2.2. Neuronal Differentiation

**Figure 1:**
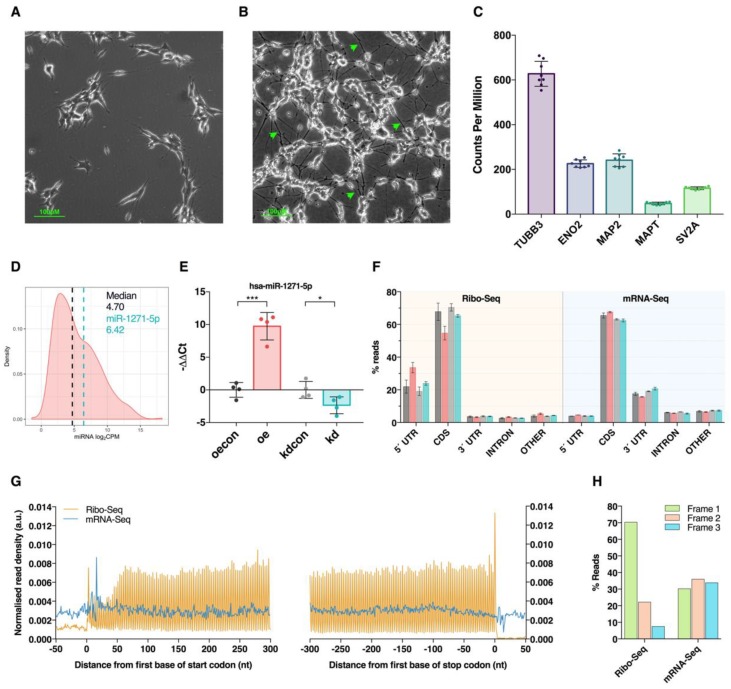
Ribosome profiling of SH-SY5Y cells after miR-1271-5p differential expression. (**A**,**B**) Phase contrast images of SH-SY5Y cells prior to (**A**) and after (**B**) treatment with retinoic acid for 5 days. Note the development of neurites (green arrowheads) after differentiation. (**C**) Expression of known neuronal marker genes in all control cells, as detected via mRNA sequencing. Data are presented as counts per million (CPM) ± SD. (**D**) Distribution of log_2_ counts per million (CPM) values for mature miRNAs expressed in differentiated SH-SY5Y cells, as detected previously by small RNA sequencing. The vertical black line represents median CPM. The vertical blue line represents miR-1271-5p CPM. (**E**) qPCR validation of miR-1271-5p overexpression (red) and knockdown (blue) after lipofection with miR-1271-5p mimic or antisense inhibitor constructs, respectively. Mimic-transfected cells were compared via a negative control scrambled siRNA (black), whereas sponge-transfected cells were compared to a negative control inhibitor construct (grey). C_t_ values were normalised to U6 and U44 housekeeping small RNAs with the geometric mean of both used as a reference. Differential expression was calculated via Student’s *t*-test with significant differential regulation of miR-1271-5p observed in both mimic-transfected cells (log_2_fc = 9.04, *p* < 0.001) and inhibitor-transfected cells (log_2_fc = −2.18, *p* < 0.05). Data presented as mean −ΔΔC_t_ ± SD. * = *p* < 0.05, *** = *p* < 0.001. (**F**) Genomic feature alignment rates for Ribo-Seq and mRNA-Seq libraries. Colour coding is the same as in Panel (**E**). (**G**) Metagene analysis of normalised read density around translation start (left) and stop (right) codons. Composite data are shown for Ribo-Seq and mRNA-Seq libraries, with ribosome-protected RNA fragments (RPFs) calibrated to predicted ribosome P sites. (**H**) Sub-codon phasing of sequencing data to each potential transcript coding sequence (CDS) reading frame revealed Ribo-Seq data that exhibits preference for the first, canonical reading frame, while mRNA-Seq data shows a relatively even spread across all three frames due to random fragmentation.

SH-SY5Y cultures were neuronally differentiated using all-trans retinoic acid (ATRA, Sigma-Aldrich, St. Louis, MO, USA). One day prior to differentiation (day −1), cells were seeded into new six well plates at a density of 25,000 cells/cm^2^ in complete culture medium. The next day (day 0), standard culture medium was replaced with ATRA-supplemented medium (10 µM final) and cells were wrapped in foil to protect from light exposure. ATRA-medium was subsequently replaced on day 2. On day 5, cells were washed with sterile PBS and returned to normal culture medium. Successful differentiation was confirmed by visual assessment of neurite outgrowth and quantification of neuronal marker genes (Figure 1A–C).

### 2.3. miR-1271-5p Transfections

Transfections were conducted 24 h after removal of ATRA-medium. Each culture was transfected with 5 µL Lipofectamine 2000 (L2K, ThermoFisher, Waltham, MA, USA) and 100 pmol of either miR-1271-5p mimic, mimic control (scrambled siRNA), an inhibitor, or an inhibitor control (mirVana, ThermoFisher). Briefly, L2K and RNA were mixed separately in 500 µL of OptiMEM (ThermoFisher) at room temperature for 5 min, after which both were combined to a final volume of 1 mL and incubated for an additional 25 min. Standard culture medium was then replaced with L2K/RNA/OptiMEM and cells were incubated at 37 °C for 6 h, following which cells were returned to normal culture medium. At 72 h post-transfection, cells were harvested as per the ribosome profiling methodology (see below) with both total RNA and ribosome protected RNA collected. In parallel to this experiment, transfection efficiency was quantified by co-transfecting additional cells with 100 pmol RNA and 2 µg of a pEZ-Lv205CT empty vector control lentiviral expression plasmid, which contains the eGFP gene under the CMV promoter. The proportion of GFP^+^ cells was analysed via Countess II FL automated fluorescent cell counter 72 h post-transfection, which revealed transfection efficiencies of: 52.5% (mimic), 75% (nonsense siRNA/mimic control), 72% (inhibitor), and 79% (inhibitor scramble control).

### 2.4. RT-qPCR Quantification of miRNA Expression

Total RNA was extracted from 300 µL of clarified lysate using 1 mL TRIzol reagent (ThermoFisher), as per the manufacturer’s instructions, with RNA precipitated at −30 °C for 2 h to enhance yield. For each sample, 750 ng of total RNA was polyadenylated with 1.25 units (U) of *E. coli* poly(A) polymerase (NEB, Ipswich, MA, USA) and 1mM ATP at 37 °C for 15 min. DNA was then degraded via the addition of 1 U DNase I (Invitrogen), after which reactions were blocked with 2.5 mM EDTA at 65 °C. Reverse transcription was subsequently performed using 200 U SuperScript II reverse transcriptase (Invitrogen) as per the manufacturer’s instructions, with 40 U RNaseOUT (Invitrogen) added to limit RNA degradation. To enhance miRNA specificity, reactions were primed with 2 µM miRNA reverse transcription primer (5′-CAGGTCCAGTTTTTTTTTTTTTTTVN-3′, where V = A, G or C and N = A, T, G, or C) as previously described [18]. Reverse transcriptase-negative reactions were additionally included, with nuclease-free water substituted for SuperScript II.

Quantitative real-time PCR reactions were then conducted using 5 µL of 1/20 cDNA dilution, 6.25 µL Power SYBR Green master mix (Applied Biosystems, Carlsbad, CA, USA), 0.5 µL of both forward and reverse primers (10 µM, see Table S1 for primer sequences), and 0.25 µL of nuclease-free water. Reactions were subsequently run on an Applied Biosystems 7500 Real Time PCR System with a dissociation curve to ensure specificity. The geometric mean of *U6* and *U44* small nucleolar RNAs was used as a reference, which was subtracted from the target gene C_t_ to determine ΔC_t_. All reactions were conducted in triplicate. Template-negative controls were run for each miRNA of interest by substituting nuclease-free water for *cDNA*. Changes in ΔC_t_ across conditions were compared via Student’s *t*-test, with *p* < 0.05 considered significant. When multiple miRNAs were tested, correction for multiple testing was performed via the Benjamini, Krieger, and Yekutieli two-stage linear step-up procedure with a false discovery rate (FDR) < 0.05 considered significant.

### 2.5. Ribosome Profiling

Ribosome profiling was conducted using the TruSeq Ribo Profile Kit (H/M/R, Illumina, San Diego, CA, USA), according to the manufacturer’s instructions with minor amendments. To inhibit translational activity, transfected cells were treated with culture medium supplemented with 0.1 mg/mL cycloheximide (CHX) for 1 min. Cells were subsequently washed with ice-cold CHX-supplemented PBS, and then harvested on ice via the addition of 1 mL mammalian lysis buffer (containing 200 µL 5 × mammalian polysome buffer, 2 µL CHX, and 10 units (U) DNase I) and extensive scraping. Ribosome foot-printing was performed by adding 90 U RNase I to 300 µL clarified lysate and incubating samples at room temperature for 45 min, after which reactions were stopped with 15 µL of a SUPERase inhibitor (Invitrogen). Ribosome-protected RNA fragments (RPFs) were then enriched using MicroSpin S-400 columns (GE Life Sciences, Marlborough, MA, USA) prior to ribosomal RNA depletion, RPF size selection, end repair, adapter ligation, reverse transcription, and PAGE purification, conducted according to the manufacturer’s instructions. RPF cDNA libraries were subsequently produced with 12 PCR cycles, after which adapter dimers were removed via 8% native polyacrylamide gel prior to library normalization and pooling. RPF libraries were then sequenced using the Illumina NextSeq 500 system, with a total of 76 single-end cycles performed. See Supplementary Methods for further details.

### 2.6. mRNA Sequencing

Total RNA was extracted from 300 µL of clarified lysate, as described above. For each sample, 1 µg of high quality (RIN ≥ 8.5) total RNA was subjected to library preparation for mRNA sequencing using the TruSeq Stranded mRNA Kit (Illumina) per the manufacturer’s instructions. mRNA was denatured (65 °C, 5 min) and ligated to oligo-dT magnetic beads (room temperature, 5 min). Afterward, unbound RNA and non-specifically bound rRNA was discarded. mRNA was then heat fragmented (94 °C, 8 min), following which reverse transcription and adapter ligation were performed as per the manufacturer’s instructions. Enrichment of cDNA fragments was subsequently conducted via 15 cycles of PCR. Afterward, libraries were normalised, pooled, and subjected to 151 cycles of sequencing using the NextSeq 500 system. See Supplementary Methods for further details.

### 2.7. Processing of Sequencing Data

Raw sequencing data were demultiplexed using *bcl2fastq2* (v2.20) with automatic adapter trimming enabled only for mRNA-Seq libraries. After merging lane data for each sample, data quality was assessed using *FastQC* (v0.11.5). Although data quality was consistently high for all samples, poor quality 3′ nucleotides (Phred33 score < 20) were trimmed using *Cutadapt* (v1.14) [19]. For Ribo-Seq data, *Cutadapt* was also used for adapter trimming, removal of single 5′ nucleotides [20], and selection of reads between 25–40 nt for further processing. Reads corresponding to rRNA, miRNA, snRNA, and snoRNA were also identified and discarded using *Bowtie2* (v2.2.6) [21]. Alignment to the reference genome (GRCh38, NCBI) was subsequently performed with *HISAT2* (v2.1.0) (mRNA-Seq) [22] or *Tophat2* (v2.2.1) (Ribo-Seq) [23] with reads aligning to features then counted via *HTSeq* (v0.7.2) [24]. Metagene analysis and sub-codon phasing were performed on alignment files using the *Plastid* python library (v0.4.8) [25]. See Supplementary Methods for further details. The data are available for download from the gene expression omnibus (GEO) with accession number GSE148827.

### 2.8. Differential Expression Analysis

Data normalisation, filtration, and differential expression analysis were performed using *EdgeR* (v3.28.0) [26]. Read-counts were first normalised to a library size (counts per million: CPM). Afterward, lowly expressed genes (5 raw counts in the smallest library) were filtered out using a minimum CPM threshold. Data was visually inspected before and after CPM filtration via multidimensional scaling (MDS) and biological coefficient of variation (BCV) plots (Figures S1 and S2). Differential expression was then calculated relative to control samples via the pairwise exact test with Benjamini-Hochberg FDR < 0.05 and absolute log_2_fc > 0.5 considered significant. Differential translational efficiency was calculated using the *RiboDiff* package (v0.2.2) [27]. See Supplementary Methods for further details and Tables S3–S5 for all differential expression results.

### 2.9. Functional Analysis

Gene ontology enrichment analysis was conducted via the *Toppfun* functional enrichment web suite [28] with Benjamini-Hochberg FDR < 0.05 considered statistically significant.

### 2.10. Analysis of miR-1271-5p Target Genes

Predicted miR-1271-5p target genes were obtained from the TargetScan database (v7.2) [12]. To analyse the effect of conserved targeting, binding site context, or site types on gene expression dynamics, genes were subcategorised as indicated in the results. For analysis of binding site types, only genes with a cumulative weighted context++ score (or total context+ score for coding sequence interactions, see below) < −0.2 were considered to limit the inclusion of false positive predictions.

miR-1271-5p binding sites located within transcript coding sequences (CDS) were predicted via TargetScan custom prediction scripts (perl), using ORF sequences for all representative human transcripts in the TargetScan database as input. For each gene, the context scores of all applicable binding sites were aggregated to produce total context+ scores as previously described [29]. For all analyses focusing on CDS binding sites, genes with 3′UTR binding sites were excluded from all comparisons, and vice versa. See Supplementary Methods for further details.

## 3. Results

### 3.1. Modulation of miR-1271-5p Expression In Vitro

To investigate the neurological function of miR-1271-5p, we bidirectionally modulated its expression in differentiated SH-SY5Y cells by transfection of either miR-1271-5p mimic or inhibitor constructs, respectively. Prior to transfection, successful neuronal differentiation of SH-SY5Y cells was confirmed by examining neurite outgrowth after retinoic acid treatment, which was later supported by the detected expression of neuronal marker genes via mRNA sequencing (mRNA-Seq; Figure 1A–C). In addition, we established that basal expression of miR-1271-5p was adequate for bidirectional modulation by examining unpublished small RNA sequencing data obtained from neuronally differentiated SH-SY5Y cells, which revealed this miRNA is within the top 36% of all expressed miRNAs (Figure 1D). Successful transfection was next confirmed by qPCR analysis with the miR-1271-5p mimic inducing significant upregulation (log_2_FC = 9.04, *p* < 0.001) compared to controls, whereas the inhibitor produced a significant downregulation (log_2_FC = −2.18, *p* < 0.05, Figure 1E). The broader impact on gene expression was then determined at both the steady state mRNA and mRNA translation level through mRNA sequencing (mRNA-Seq) and ribosome profiling (Ribo-Seq), respectively. mRNA-Seq and Ribo-Seq produced an average of 61.53 × 10^6^ (±24.17 × 10^6^) and 39.30 × 10^6^ (±5.53 × 10^6^) raw reads per sample, respectively, of which 57.58 × 10^6^ (±22.57 × 10^6^) and 8.12 × 10^6^ (±3.25 × 10^6^) were retained after quality control and genome alignment (Table S2).

While mRNA-Seq reads exhibited preference for coding sequence (CDS, ~61%) and 3′UTR (~17%) alignment, and showed even read density across CDS start/stop codons and reading frames due to random fragmentation (Figure 1F–H), the Ribo-Seq libraries exhibited hallmark features of mRNA translation consistent with successful enrichment of ribosome protected RNA fragments (RPFs). Analysis of genome alignment first revealed RPFs predominantly mapped to mRNA CDS (~60%) and 5′UTR (~23%) regions, which reflected the expected location of actively translating ribosomes (Figure 1F). Further inspection of RPF metagene alignment reached a maximal density between CDS start and stop codons, with characteristic peaks indicative of translational initiation and termination (Figure 1G). RPF sub-codon phasing also displayed a clear preference for CDS reading frame 1 (70% of RPFs), which reflected the triplet periodicity of ribosomal translocation (Figure 1H).

### 3.2. Transcriptome-Wide Remodelling Induced by miR-1271-5p Differential Expression

**Figure 2:**
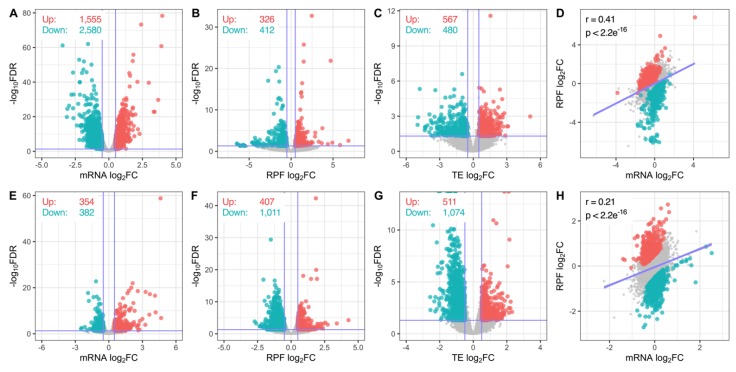
Differential expression analysis and functional annotation. (**A**–**C**) Volcano plots comparing gene log_2_ fold-change and –log_10_ false discovery rate (FDR, Benjamini-Hochberg) at the mRNA (**A**), RPF (**B**)**,** and TE (**C**) levels after miR-1271-5p overexpression. Horizontal line represents FDR = 0.05. Significantly upregulated and downregulated genes (FDR < 0.05, log_2_fc > | ± 0.5|) are marked in red and blue, respectively. (**D**) Comparison of gene mRNA and RPF log_2_ fold-change expression values. Pearson’s correlation coefficients and associated *p*-values reported top left. Coloured genes were subjected to significant changes in TE. (**E**–**H**) As in (**A**–**D**), except after miR-1271-5p knockdown.

Differential expression analysis revealed miR-1271-5p overexpression significantly altered 4135 genes at the mRNA level (1555 upregulated, 2580 downregulated) and a further 738 genes (326 upregulated, 412 downregulated) at the RPF level (Figure 2A,B). When changes in RPF expression were scaled to mRNA levels by analysing translational efficiency (TE), 1047 genes (567 upregulated, 480 downregulated) were identified as significantly regulated (Figure 2C). Direct comparison of mRNA and RPF expression changes further revealed these factors were positively correlated (Pearson’s r = 0.41, *p* < 2.2 × 10^−16^), which indicated modulation of mRNA abundance and translation was generally proportional (Figure 2D).

Gene expression changes were more strongly affected at the translational level after miR-1271-5p knockdown with 736 genes (354 upregulated, 382 downregulated) regulated at the mRNA level and 1,418 (407 upregulated, 1011 downregulated) regulated at the RPF level (Figure 2E,F). This was further reflected in the identification of 1585 genes (511 upregulated, 1074 downregulated) with significantly altered TE (Figure 2G). The correlation between mRNA and RPF expression was reduced (r = 0.21, *p* < 2.2 × 10^−16^) after miR-1271 knock down compared to overexpression, which suggests a reduction in the miRNA led to uncoupling of translation from mRNA abundance (Figure 2H).

### 3.3. Impact of Conserved Targeting and Binding Site Context on mRNA Repression

To explore the mechanistic basis of target mRNA repression, we next analysed the expression profiles of all mRNAs with predicted miR-1271-5p binding sites in the 3′UTR (TargetScan v7.2), through which miRNA are thought to canonically function [11]. We reasoned that, if miR-1271-5p primarily regulates mRNA abundance, modulation of target genes would be observed at the mRNA and RPF levels, whereas prevalent translational regulation would result in changes in RPF expression only. To additionally account for the effects of conserved targeting and binding site accessibility, interactions were further stratified by the probability of conserved targeting (P_ct_) or a cumulative weighted context++ score (CWCS) [29,30].

**Figure 3:**
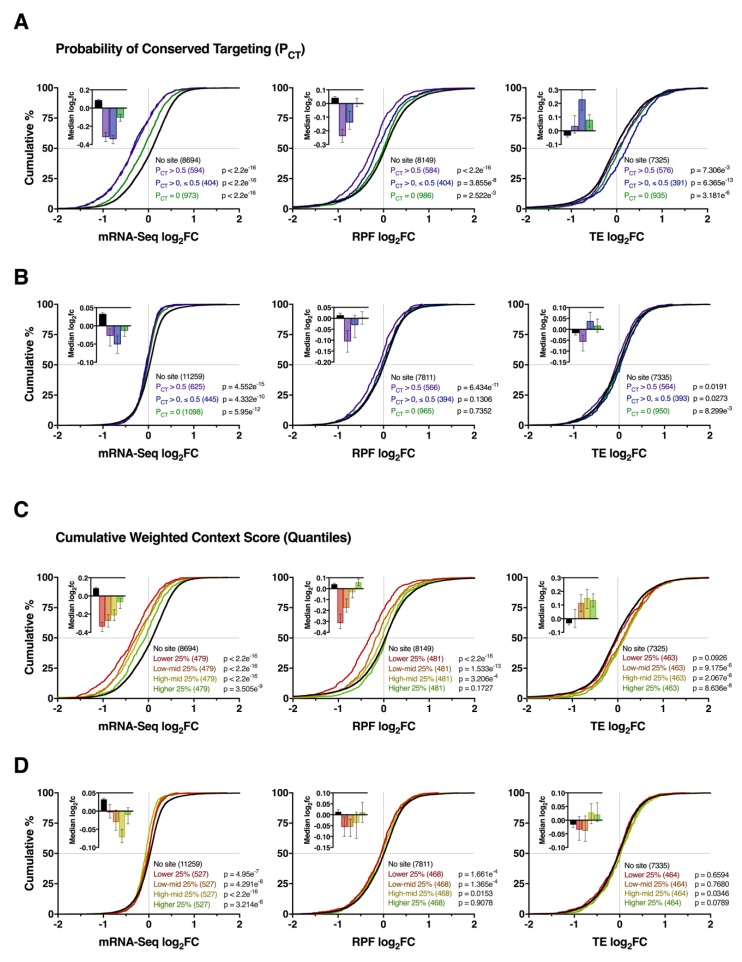
Effect of conserved targeting and binding site context on miR-1271-5p-mediated repression. **(A)** Cumulative distribution of mRNA, RFP, and TE log_2_ fold changes for target genes with 3′UTR miR-1271-5p binding sites after overexpression. Using the TargetScan probability of the conserved targeting (P_ct_) metric [31], genes were stratified into highly conserved (P_ct_ > 0.5), lowly conserved (P_ct_ ≤ 0.5, > 0), or non-conserved groups (P_ct_ = 0) to examine the effect of conserved targeting. P values were calculated via the two-sided Kolmogorov-Smirnov test versus genes with no predicted binding sites. Median log_2_ fold changes ± 95% CI are reported top left. (**B**) as in (**A**), except after miR-1271-5p knockdown. (**C**,**D**) As in (**A**,**B**), except after analysing the effect of binding site context on repression efficacy. For this analysis, target genes were stratified into quartiles based on the cumulative weighted context++ score (CWCS), wherein lower scores correspond to more favourable interactions [29].

As expected, miR-1271-5p overexpression was associated with downregulation of its predicted mRNA targets (*p* ≤ 3.505 × 10^−9^) and their RPFs (*p* ≤ 2.522 × 10^−3^ for all groups, except the upper quartile of genes after stratification via CWCS) compared to genes without a predicted interaction (Figure 3A,C). Moreover, we observed a hierarchal effect on repression of targets stratified by P_ct_ and CWCS such that genes with higher P_ct_ and lower CWCS (i.e., more favourable binding site context) were repressed with the greatest magnitude (Figure 3A,C). Furthermore, while changes in mRNA and RPF expression were correlated for all groups (Pearson’s r ≥ 0.38, *p* < 2.2 × 10^−16^, Figure S3), RPF downregulation was slightly less pronounced, which resulted in consistently upregulated TE for most groups of target genes (Figure 3A,C). Overall, these results support the mRNA destabilisation scenario with conserved targeting and binding site context contributing to repression efficacy.

By contrast, the regulation of target genes after miR-1271-5p knockdown was comparatively modest, with little discernible influence from target site conservation or binding site context detected in gene distribution profiles (Figure 3B,D). Most of the target gene expression changes were restricted to the mRNA level, in which all groups exhibited a significant, albeit modest, bias towards downregulation relative to genes with no target site, contrary to expectation (Figure 3B,D). RPF and TE level changes were largely inconsistent in comparison (Figure 3B,D), while correlations between mRNA and RPF expression changes were weak relative to the overexpression experiment (r ≤ 0.24, *p* ≤ 0.024, Figure S3). Together, these findings suggest miR-1271-5p knockdown produced minimal changes in target gene expression profiles with conserved targeting and binding site context exhibiting no major effect.

### 3.4. Effect of Seed Complementarity on Target mRNA Expression

**Figure 4:**
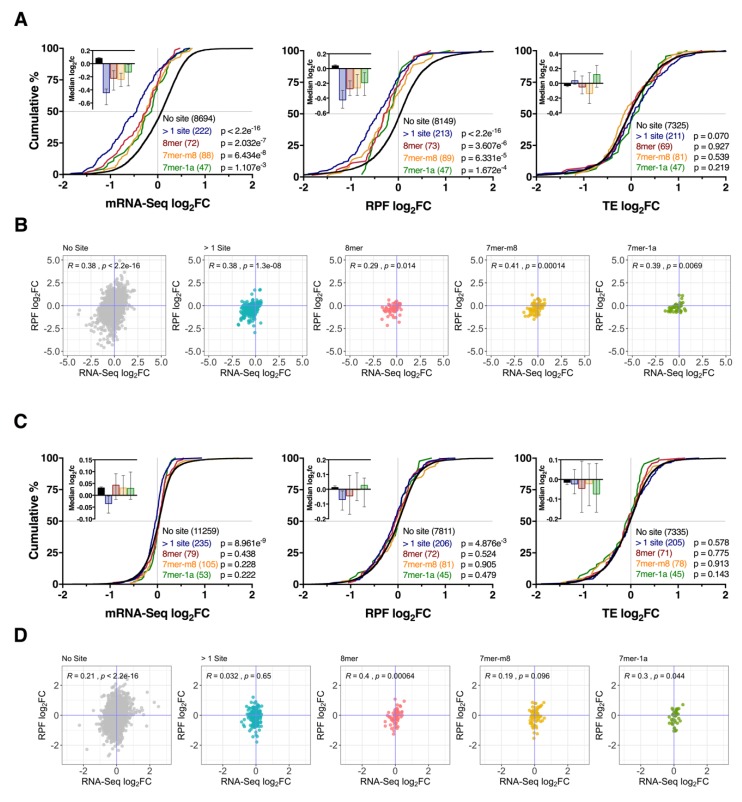
Seed-mRNA complementarity affects the magnitude of repression. (**A**) Cumulative distribution of mRNA, RFP, and TE log_2_ fold changes after miR-1271-5p overexpression for genes with multiple 3′UTR binding sites, or single 8mer, 7mer-m8, or 7mer-1a sites. For this analysis, interactions with cumulative weighted context++ score < −0.2 were investigated to limit the inclusion of false positive miRNA-mRNA pairings, while retaining non-conserved interactions with potentially high importance. *p*-values were calculated relative to genes with no sites via the two-sided Kolmogorov-Smirnov test. Median log_2_ fold-changes ± 95% CI reported top left. (**B**) Scatter plots comparing mRNA and RPF log_2_ fold changes for genes analysed in panel (**A**), with Pearson’s correlation coefficients and p values reported top left. (**C**,**D**) As in (**A**,**B**), except after miR-1271-5p knockdown.

We next examined whether seed-mRNA base pairing influenced the strength of mRNA repression. While target mRNA and their RPFs displayed a tendency for downregulation after miR-1271-5p overexpression (*p* ≤ 1.107 × 10^−3^ and *p* ≤ 1.672 × 10^−4^, respectively), the magnitude of repression was also consistent with the relative order of seed strength (> 1 site > 8mer > 7mer-m8 > 7mer-1a, Figure 4A). The reduction of RPFs was also correlated with the change in steady state mRNA levels (r ≥ 0.29, *p* ≤ 0.014, Figure 4A,B) for all target genes with no significant changes in gene TE (*p* ≥ 0.07). Analysis of target genes after miR-1271-5p knockdown again revealed no clear pattern across all three datasets (Figure 4C). While mRNA-RPF ratios for 8mer (r = 0.4, *p* = 6.4 × 10^−4^) and 7mer-1a (r = 0.3, *p* = 0.044) interactions were correlated, genes with >1 site (r = 0.032, *p* = 0.65) or single 7mer-m8 (r = 0.19, *p* = 0.096) sites showed no association, which indicates seed-mRNA complementarity exhibited no consistent effect on mRNA-RPF ratios after miR-1271-5p knockdown (Figure 4D).

### 3.5. Repression of mRNA via Binding Sites within the Coding Sequence

**Figure 5:**
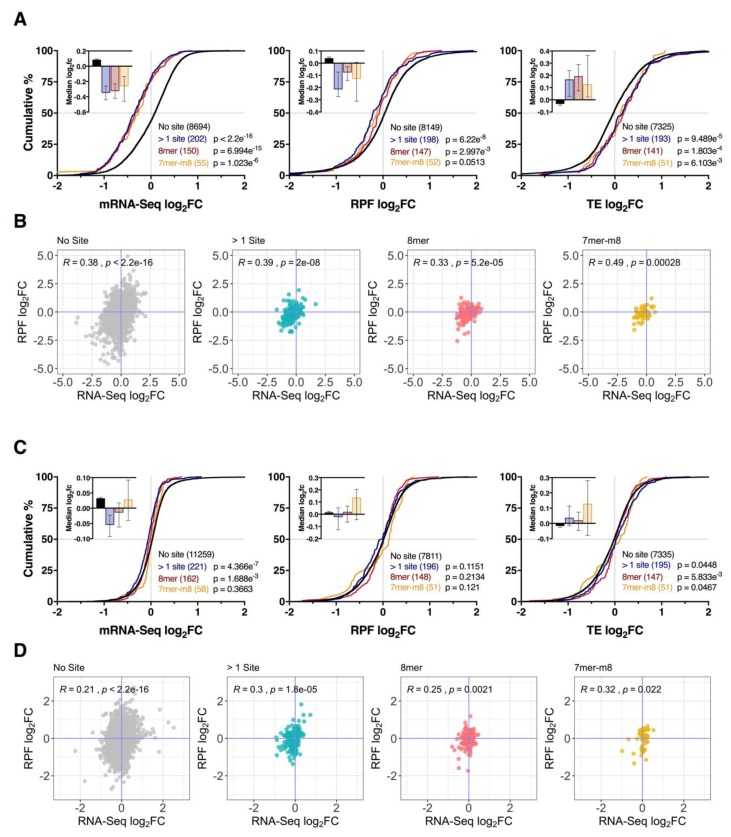
Coding sequence binding sites function similarly to 3′UTR sites. Potential miR-1271-5p binding sites housed within mRNA coding sequences were predicted using custom prediction scripts provided by TargetScan with interactions exhibiting a total context+ score < −0.2 retained for further analysis. (**A**) Cumulative distribution analysis of mRNA, RPF, and TE expression for target genes with predicted CDS sites after miR-1271-5p overexpression. Note that, for this analysis, no 7mer-1a interactions passed context score filtration. Groups were compared to genes with no site via two-sided Kolmogorov-Smirnov test. Median log_2_ fold-changes ±95% CI reported top left. (**B**) Scatter plots comparing mRNA and RPF log_2_ fold changes for genes analysed in the panel (**A**) with Pearson’s correlation coefficients and p values reported in the top left. (**C**,**D**) As in (**A**,**B**) except after miR-1271-5p knockdown.

In previous studies, miRNA binding sites within the mRNA coding sequence (CDS) have demonstrated capacity to repress active translation while preserving mRNA stability [32,33,34]. To determine if miR-1271-5p employs such a mechanism, we used the TargetScan algorithm to identify all mRNAs with predicted CDS binding sites. In total, 3367 representative transcripts with at least 1 CDS binding site (and no 3′UTR sites) were identified, of which 590 returned a total context+ score (TCS) < − 0.2. In miR-1271-5p overexpressing cells, we found that CDS binding sites consistently decreased mRNA levels (*p* ≤ 1.023 × 10^−6^). However, the magnitude of repression was unaffected by seed-type (Figure 5A). Downregulation of RPF expression was comparatively weak for all groups, which results in a systematic increase in TE (*p* ≤ 6.103 × 10^−3^). This suggests CDS binding sites result in stronger mRNA degradation than translational repression (Figure 5A). Analysis of mRNA-RPF ratios further supported the mRNA destabilisation hypothesis, with positive correlations identified for all seed types (r ≥ 0.33, *p* ≤ 2.8 × 10^−4^, Figure 5B). Although knockdown of miR-1271-5p resulted in fold change distributions comparable to 3′UTR analyses, we found that all seed types exhibited positive mRNA-RPF correlations, which suggests that, while biases in target gene expression were weak, changes in mRNA abundance and translation were generally consistent (Figure 5C,D).

The possibility for synergistic effects between 3′UTR and CDS binding sites was also explored in genes expressing both target site contexts. Overall, a robust loss of mRNA abundance (*p* = 1.227 × 10^−11^) and translation (*p* = 1.17 × 10^−4^) was observed after miR-1271-5p overexpression, which suggests the combination of both results in highly effective gene repression (Figure S4). Repression of mRNA abundance (*p* = 1.319 × 10^−4^) and translation (*p* = 7.786 × 10^−3^) was also observed after miR-1271-5p knockdown. However, there was no change in TE or mRNA-RPF ratios (Figure S4).

### 3.6. miR-1271-5p Knockdown Triggers Upregulation of miRNAs with Similar Seed Sequence

**Figure 6:**
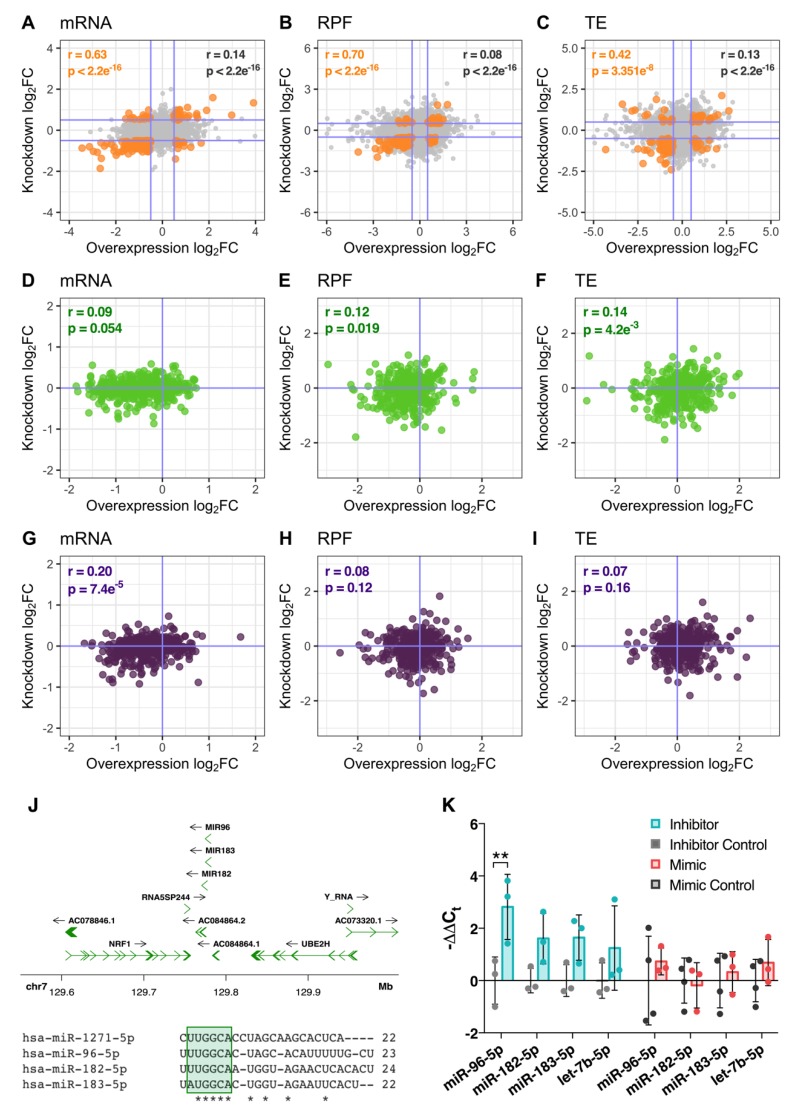
Comparison of gene expression profiles between experiments and analysis of miRNAs with a similar seed sequence. (**A**–**C**) Scatter plots comparing mRNA (**A**), RPF (**B**), and TE (**C**) log_2_ fold changes between overexpression and knockdown conditions. Genes significantly regulated in both conditions are marked in orange. All other genes are coloured grey. (**D**–**I**) As in (**A**–**C**), except examining the expression of genes with only 3′UTR (**D**–**F**) or CDS (**G**–**I**) miR-1271-5p binding sites. (**J**) Genomic context of the miR-96/183/182 polycistronic miRNA cluster and multiple sequence alignment versus miR-1271-5p. Note the similar seed regions, shaded in green. Asterisks denote bases conserved amongst all 4 miRNAs. (**K**) qPCR analysis of miR-96-5p, miR-182-5p, and miR-183-5p expression levels after miR-1271-5p knockdown and overexpression. let-7b-5p was also included to ensure any changes in the miR-196/183/182 cluster were not due to global rises in miRNA processing. Data presented as mean −ΔΔC_t_ ± SD. Expression was compared to control cells via Student’s *t*-test with post-hoc correction for multiple testing using Benjamini, Krieger, and the Yekutieli method. FDR < 0.05 was considered significant (** = FDR < 0.01).

While miR-1271-5p overexpression produced a distinct profile of decreased target gene expression that was consistent with expectation, we were surprised to also observe a decrease in predicted target expression when the miRNA was repressed. In other words, genes significantly regulated under both conditions were found to be positively correlated at the mRNA level (Pearson’s r = 0.63, *p* < 2.2 × 10^−16^, 193 genes), their RPFs (r = 0.70, *p* < 2.2 × 10^−16^, 136 genes), and associated TE (r = 0.42, *p* = 3.35 × 10^−8^, 163 genes, Figure 6A–C). Similarly, target genes with 3′UTR and CDS localised miRNA binding sites were also positively correlated for both treatments at the RFP (r = 0.12, *p* = 0.019) and (r = 0.2, *p* = 7.4 × 10^−5^), respectively (Figure 6D–I). Collectively, these observations suggested the miRNA knockdown was somehow enhancing the function of its target miRNA rather than suppressing it.

In view of emerging research suggesting that changes in miRNA expression can induce compensatory patterns of expression in related miRNAs with a highly similar sequence [35,36], we examined the expression of miRNAs derived from an intergenic cluster encoding miR-96/183/182, located within the 7q32.2 locus (Figure 6J). These miRNAs have identical or highly similar seed regions to miR-1271-5p, and, therefore, display a very substantial overlap in their pool of predicted target genes (Figure 6J). In support of this hypothesis, miR-96-5p was significantly upregulated after miR-1271-5p knockdown (log_2_fc = 2.78, FDR = 0.009), whereas miR-182-5p (FDR = 0.065), miR-183-5p (FDR = 0.065), and an unrelated miRNA for comparison, let-7b-5p (FDR = 0.111), showed no significant change (Figure 6K). No change in miR-96-5p was detected after miR-1271-5p overexpression, which suggests that miR-96-5p was specifically induced in response to miR-1271-5p suppression (Figure 6K).

### 3.7. Functional Analysis of Differentially Regulated Genes

GO term analysis of significantly regulated genes revealed a clear functional division between upregulated and downregulated genes in both conditions. After miR-1271-5p overexpression, genes upregulated at the RPF level were heavily enriched for GO terms related to DNA and chromatin organisation, with *chromosome organisation*, *DNA packaging complex*, and *nucleosome* among top enriched ontologies (Table S6). In contrast, GO terms associated with regulation of the extracellular matrix and cellular locomotion were considerably represented in downregulated genes, including *collagen-containing extracellular matrix*, *cell migration*, and *cell motility* (Table S6). Overall, these findings were largely consistent with analyses conducted on genes with significantly regulated mRNA levels and TE. However, genes with downregulated TE exhibited minimal to no enrichment (Tables S7 and S8).

Knockdown of miR-1271-5p produced functionally distinct translational profiles relative to the overexpression experiment. Specifically, upregulated genes were heavily enriched for cellular component and biological process terms associated with mitochondria, such as *mitochondrion*, *mitochondrial inner membrane*, and *mitochondrial protein complex* (Table S6). Although downregulated genes were overrepresented for a variety of terms related to post-translational protein modifications and wnt signaling, we also found enrichment of terms related to neuronal development among top ontologies, including *neuron projection development*, *neuron development*, and *neurogenesis* (Table S6). These findings differed considerably from the mRNA level in which upregulated genes were predominantly representative of DNA regulation while downregulated genes were enriched for terms related to the extracellular matrix and cellular locomotion (Table S7). Analysis of genes with altered TE, however, revealed that downregulated genes exhibited similar GO term enrichment relative to the RPF level (Table S8).

### 3.8. Functional Analysis of Genes Directly Targeted by miR-1271-5p

**Figure 7:**
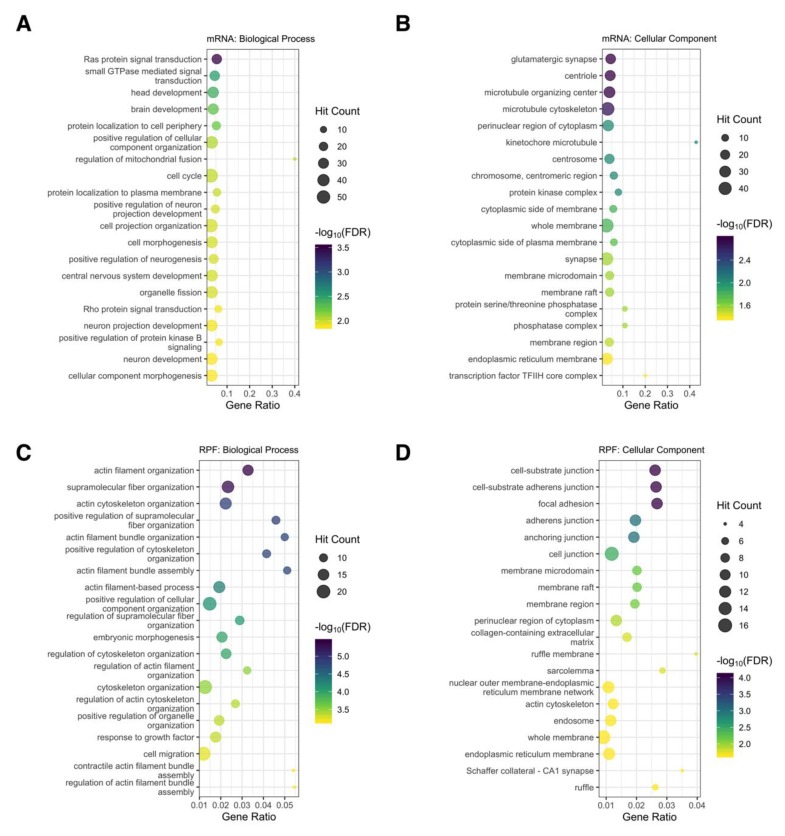
Functional analysis of directly targeted genes. (**A**) Top 20 significantly enriched biological process gene ontology terms after analysing target genes significantly downregulated at the mRNA level in the overexpression experiment. Note that the gene ratio refers to the overlap between the number of genes in the query versus the number of genes in the gene ontology term. (**B**) As in (**A**), except for depicting the top 20 cellular component gene ontologies. (**C**,**D**) As in (**A**,**B**), except after analysing target genes significantly downregulated at the RPF level.

With mRNA degradation identified as the predominant mechanism employed by miR-1271-5p, we next examined the functional composition of directly targeted genes. Since miR-1271-5p overexpression consistently produced the most pronounced effect on target gene expression, functional analysis was conducted on target genes (CWCS/TCS < −0.2) significantly downregulated in this condition (see Table S9 for a full list of genes). At the mRNA level (328 genes, interaction summary presented in Figure S5A), 112 biological process and 44 cellular component ontologies were significantly enriched with no overall bias in functional representation (Figure 7A,B). *Positive regulation of neuron projection development*, *neuron development*, *glutamatergic synapse*, and *synapse* terms were found to be enriched, which suggests that, while miR-1271-5p target genes are functionally diverse, a subset appear to be involved in neuronal function (Figure 7A,B, Table S9). When this analysis was extended to differentially translated genes (81 genes, interaction summary presented in Figure S5B), 186 biological process annotations were identified with strong representation of processes relating to the actin cytoskeleton, including *actin filament organization*, *actin cytoskeleton organization*, and *actin filament bundle assembly*, among others (Figure 7C, Table S10). Furthermore, cellular component ontologies relating to cellular junctions, such as *cell-substrate junction*, *focal adhesion*, *adherens junction*, and *anchoring junction* were enriched (Figure 7D, Table S10). While these terms relate to synaptic function, we also observed transcriptional (*GPHN*, *GRM7*, *RGS2*, *SLC1A1*, *GRID1*, *SLC12A5*, and *HOMER1*) and translational (*GPHN*, *GRM7*, *RGS2*, *SLC1A1*, and *HOMER1*) downregulation of genes involved in neuronal differentiation and neurotransmission, previously identified as miR-1271-5p targets by Jensen and Covault [15] (Figure S6). We also examined the expression of *FOXK2*, which has previously exhibited reciprocal expression with miR-1271-5p in hepatocellular carcinoma tissue, but we were unable to replicate this in SH-SY5Y cells [37]. Similarly, while *ZIC2* was previously shown to be modulated by the miRNA [38], it was not expressed in differentiated SH-SY5Y cells.

## 4. Discussion

Molecular interventions or perturbations of neuronal miRNAs in model systems is an important strategy for understanding the impact of their dysregulation on gene expression networks in psychiatric disorders. In the current study, we implemented parallel mRNA sequencing and ribosome profiling to explore the impact of the schizophrenia-associated miR-1271-5p [16] on neuronal gene expression and determine the mechanistic basis of its regulatory function. This revealed that differential expression of miR-1271-5p not only alters the abundance and translation of mRNAs with applicable binding sites, but also considerably disrupts the transcriptome as a whole. 

miRNAs are generally thought to simultaneously reduce translational capacity and stability of target mRNAs by binding 3′UTR sequences and inducing sequential deadenylation (CCR4-NOT and PAN2-PAN3 complexes), de-capping (DCP2 and associated factors) and 5′ to 3′ degradation (XRN1 exoribonuclease) [11]. Our observations were broadly supportive of this hypothesis with mRNAs containing miR-1271-5p binding sites within the 3′UTR being repressed predominantly via mRNA destabilisation after miR-1271-5p overexpression. This is consistent with the canonical model of miRNA function [11] and is supported by previously observed miRNA-mRNA interaction dynamics after analysis of ribosome protected RNA fragments [39,40,41]. With regard to the smaller subset of miRNA-mRNA interactions that directly modulate translation (such as *TMEM52*, *SEPT5*, and *MARCH3*), we suspect they are highly context-specific and may involve other post-transcriptional regulatory elements or RNA modifications, such as m6A methylation [42]. However, it could be argued that the time-frame between miR-1271-5p transfection and cell harvest may have precluded the detection of some early translational repression. Regardless of the mode of action, we observed consistent effects of target site conservation, binding site context, and seed-types on repression efficacy, which supports the effectiveness of these features for predicting miRNA-mRNA pairings with high effect size in silico [12,29,31].

We observed a similar effect of miR-1271-5p on mRNAs with binding sites localized within the open reading frame, which was characterized by suppression of both mRNA steady state abundance and translation. However, in this case, the effect was indifferent to seed the binding parameters that were a feature of target sites contained in 3′UTR segments. Nonetheless, these findings suggest that miRNA binding sites within open reading frames are functionally significant, which support other observations of CDS-miRNA interaction in metazoans [43]. Additional studies have further strengthened the view that open reading frame target sites are employed to some extent by miRNA for mRNA regulation [44,45,46]. Unlike 3′UTR sites, however, no canonical model of miRNA repression within coding segments has been established to date. In a variety of cellular contexts, recent studies have shown that a subset of miRNAs—including miR-29, miR-181, miR-15a/miR-16, and miR-92a—decrease the stability of target genes through CDS binding sites, which supports our findings for miR-1271-5p [44,45,46]. Other lines of evidence suggest that CDS sites are predominantly used for translational repression with ribosome stalling or ribosome drop-off proposed as the mechanism of repression [32,33,34]. Our data opposes this hypothesis, as genes with miR-1271-5p CDS binding sites were found to be more effectively repressed at the mRNA level when compared to the RPF level. This further suggests that translationally active mRNAs may possess a degree of resistance to miRNA-mediated repression. Considering these observations together, further investigation into the true functional relevance of CDS-located miRNA binding sites is required to elucidate the nature of these binding sites and determine their biological significance in the neuronal context.

When we considered the target gene ontologies at a transcriptome-wide level, we observed that overexpression of miR-1271-5p predominantly affected genes involved in DNA organisation, extracellular matrix interactions, and cellular locomotion, while knockdown impacted genes associated with mitochondria, post-translational protein modifications, wnt signaling, and neuronal function. This suggests the functional impact of miR-1271-5p bi-directional regulation is not inverse in nature. While these pathways are significant to neural function and synaptic connectivity, the extracellular matrix is of particular interest with regard to psychiatric disorders, such as schizophrenia, as components of these systems, including reelin (RELN), are known to participate [47,48]. We observed that this gene encoding an extracellular matrix glycoprotein involved in the regulation of neuronal migration in the developing brain was repressed at both the mRNA and translation level in miR-1271-5p transfected cells. We also observed strong enrichment of miR-1271 regulated genes in ontologies relating to actin filament dynamics and cellular junctions. Both of these mechanisms are crucial for regulation of neuronal morphology, neural circuits, and the synaptic response to neural activity. These gene networks are particularly significant in the context of psychiatric disease given that downregulation of miR-1271-5p has been reported in cohorts of individuals with schizophrenia [16,17]. Furthermore, these results considerably extend previous findings, which reported that miR-1271-5p regulates a small number of genes involved in neuronal differentiation and neurotransmission [15]. We can make some further assumptions about the role of miR-1271-5p in the brain by looking at the activity of its seed pairing homolog miR-96-5p, as it is likely they have similar targets and regulatory activity [15]. For example, miR-96-5p expression has been associated with the coordination of neural crest [49] and auditory hindbrain development [50], as well as the induction and progression of neuronal differentiation [51,52,53]. It has also been shown to be involved in the regulation of long-term memory [54,55] and pain sensitivity [56]. Furthermore, mutations in the miR-96-5p seed region have been linked to progressive hearing loss due to defects in the cochlear hair cells [57,58]. Given these findings, more investigation into the effects of miR-1271-5p on brain and behaviour are warranted and may further highlight its significance to the pathophysiology of psychiatric disorders. 

Although miR-1271-5p overexpression produced defined patterns of target gene repression, we were unable to identify any consistent trends after miR-1271-5p knockdown. In addition, knockdown-induced gene expression profiles were found to be positively correlated with the overexpression experiment when significantly regulated genes and target genes were analysed, which implies a degree of concordance between experimental conditions. While these findings did not conform with dynamics observed in previous miRNA studies [39,40,41], further investigation revealed miR-96-5p was upregulated after miR-1271-5p knockdown, which suggests miR-1271-5p loss induces a compensatory response. Considering these miRNAs share identical seed regions, exhibit high sequence homology, and are thought to repress similar target gene networks [15], we suspect upregulation of miR-96-5p contributed to the observed patterns of knockdown-induced gene expression. Additional confirmation is required to identify whether miR-182-5p and miR-183-5p were also responsive when considering these miRNAs are also processed from the polycistronic miR-96/183/182 transcript [59] and approached statistically significant upregulation in our data. Regardless, this remains a particularly interesting finding in the current study. While no studies to date have identified evidence for a compensatory response between miR-1271-5p and miR-96-5p, such a mechanism is not unprecedented in the literature. For the miR-449 and miR-34 families, which share the same seed region, homozygous deletion of one family has been shown to increase expression of the other in mouse models [35,36]. In the neuronal context, functional redundancy among the polycistronic miR-96/183/182 cluster has also been described through phenotypic studies. Specifically, homozygous knockout of miR-182 in the mouse retina has previously been reported to induce no discernible phenotype, which suggests miR-96 and miR-183 compensate for miR-182 loss [60]. Likewise, while miR-96/182/183 CRISPR/Cas9 mutants compromised zebrafish hair cells, individual and combined miR-96/183 and miR-183/182 mutants had a limited effect, which further suggests functional redundancy among these miRNAs [61]. It would, therefore, be interesting to determine whether this redundancy extends to miR-1271-5p, considering the miR-196/183/182 cluster is involved in hippocampal long-term memory [54], pain sensitivity [56,62], retinal cell function [60], and hair cell maintenance [61], while dysregulation has been identified in a range of neurological and psychiatric disorders [59].

In summary, our findings illustrate the substantial disruption of post-transcriptional dynamics that ensues the following differential expression of miR-1271-5p in the neuronal context. Since this miRNA appears to fulfill regulatory roles associated with extracellular matrix interactions, cytoskeletal regulation, and neuronal function, we suspect dysregulation of this miRNA in schizophrenia contributes to the disorder. In addition, our data provides compelling support for the hypothesis that miRNA predominantly repress mRNA abundance through 3′UTR and CDS recognition motifs. However, further miRNA profiling studies are required to determine the full extent of miRNA function in the neuronal context and assess the contribution of other regulatory systems.

## Supplementary Materials

The following are available online at https://www.mdpi.com/2073-4409/9/4/1014/s1, Supplementary Methods. Figure S1: Multidimensional scaling (MDS) plots. Figure S2: Biological coefficient of variation (BCV) plots. Figure S3: Relationship between miR-1271-5p target mRNA abundance and translation. Figure S4: Combined effect of 3′UTR and CDS miR-1271-5p binding sites on target mRNA expression. Figure S5: miR-1271-5p target genes interaction networks. Figure S6: Differential expression of previously identified miR-1271-5p target genes. Table S1: qPCR Primer Sequences. Table S2: Read Processing Statistics. Table S3: mRNA Level Differential Expression Analysis. Table S4: RPF Level Differential Expression Analysis. Table S5: TE Level Differential Expression Analysis. Table S6: Functional Analysis of Genes Regulated at the RPF Level. Table S7: Functional Analysis of Genes Regulated at the mRNA Level. Table S8: Functional Analysis of Genes with Significantly Altered Translational Efficiency. Table S9: List of Significantly Downregulated miR-1271-5p Target Genes. Table S10: Functional Analysis of Significantly Regulated miR-1271-5p Target Genes After Overexpression.
