# Supplementary material for: Characterising the Transcriptional and Translational Impact of the Schizophrenia-Associated miR-1271-5p in Neuronal Cells
Source: Cells. 2020 Apr 18;9(4):1014. doi: 10.3390/cells9041014 (PMC7226585; doi:10.3390/cells9041014)
Supplement: Supplementary file 1 [file cells-09-01014-s001.zip › cells-761301.revised.manuscript.supp.material/mir.1271.supp.figs.docx]

**SUPPLEMENTARY FIGURES S1–6**

**
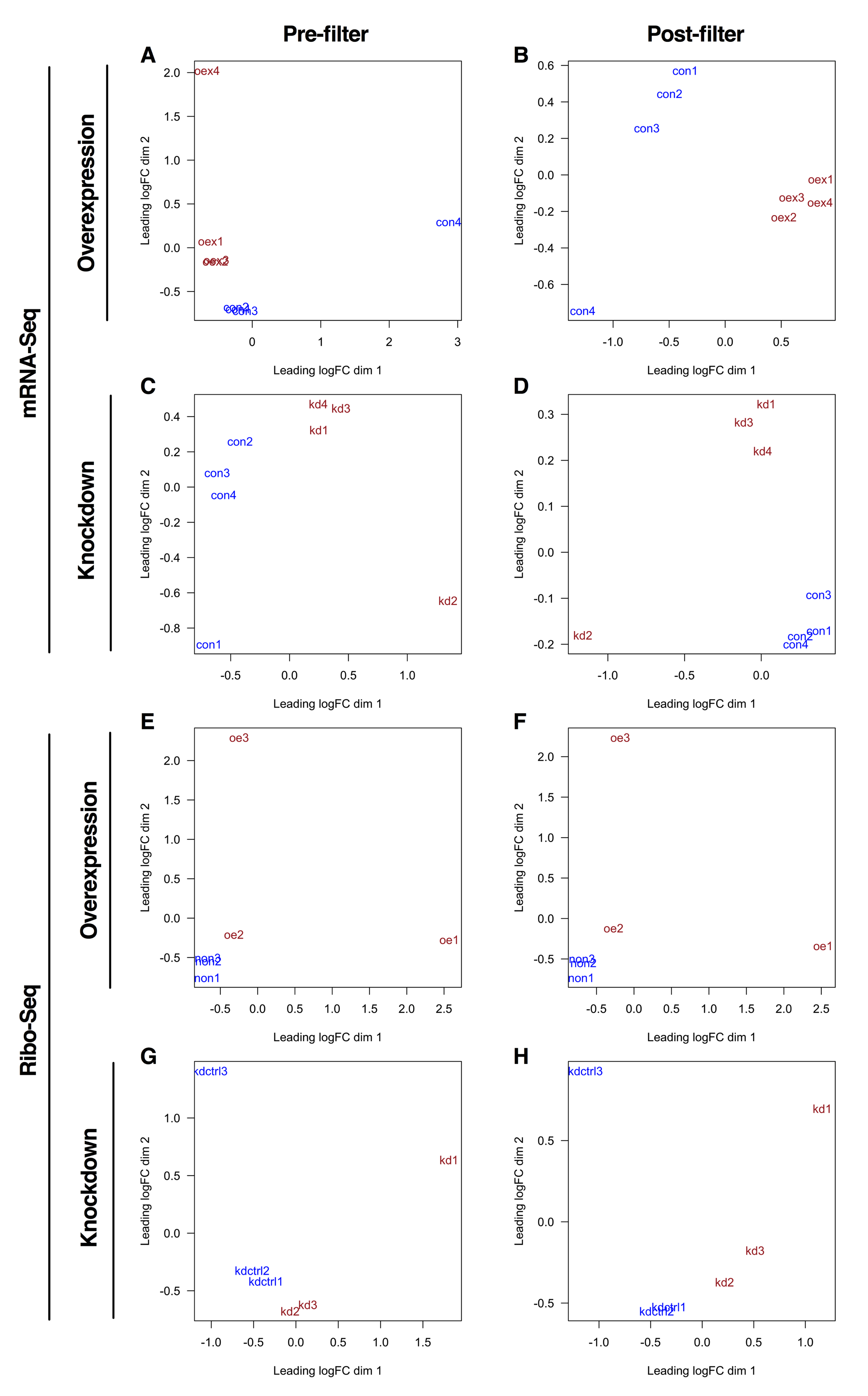
**

**Supplementary Figure 1. Multidimensional scaling plots.** Multidimensional scaling (MDS) plots of mRNA-Seq **(A–D)** and Ribo-Seq **(E–H)** read-count data before **(A, C, E, G)** and after **(B, D, F, H)** removal of low read-count genes. In general, control (blue) and treated (red) samples clustered more tightly after CPM filtration.


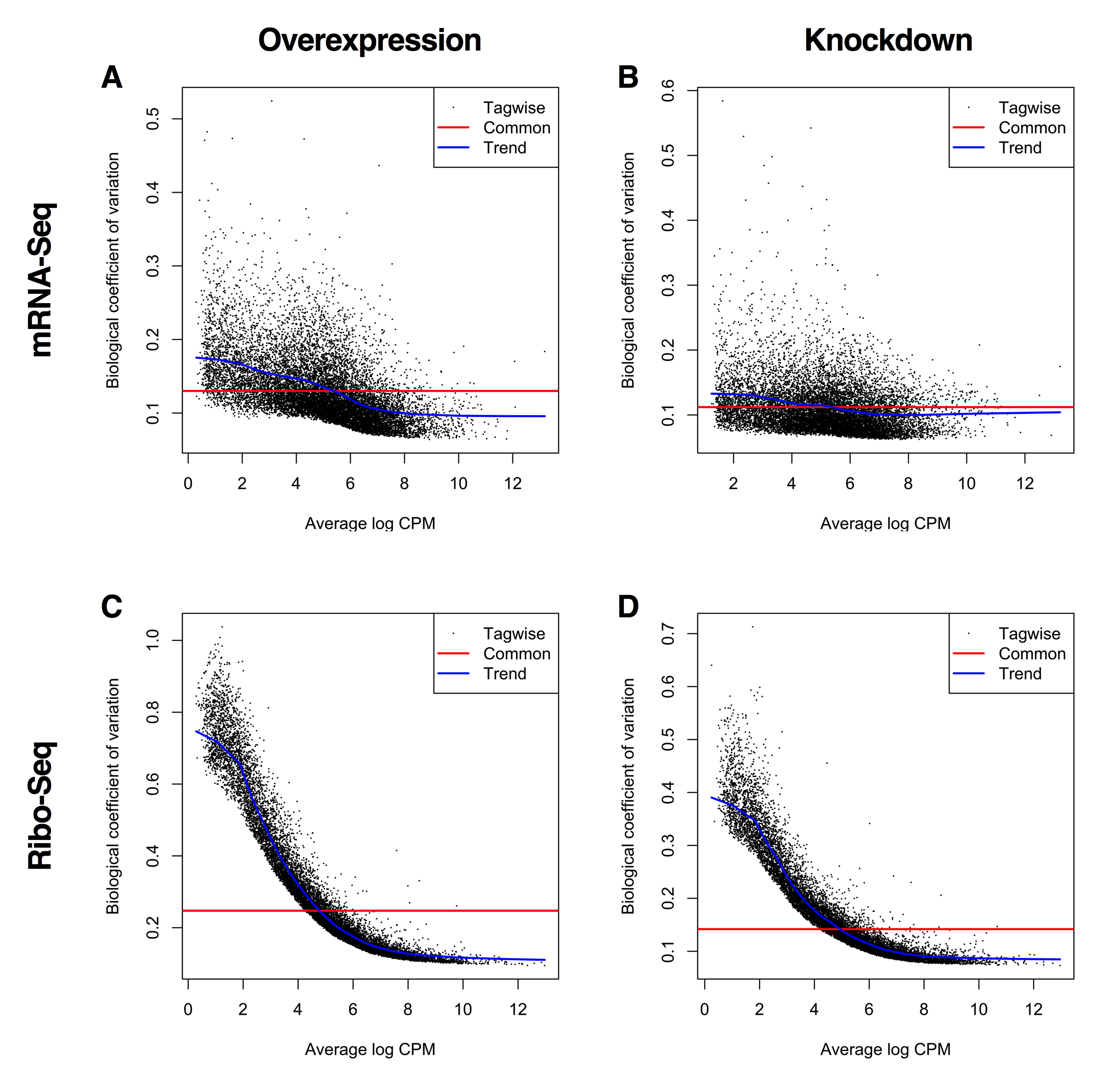


**Supplementary Figure 2. Biological coefficient of variation plots.** Biological coefficient of variation (BCV) plotted against average log_2_ counts per million for all genes in the mRNA-Seq **(A & B)** and Ribo-Seq **(C & D)** data sets for the miR-1271-5p overexpression **(A & C)** and knockdown **(B & D)** conditions. The blue trend lines indicate that biological variation was most prominent in low-read genes. Higher variation is seen in the Ribo-Seq data due to smaller sample size.


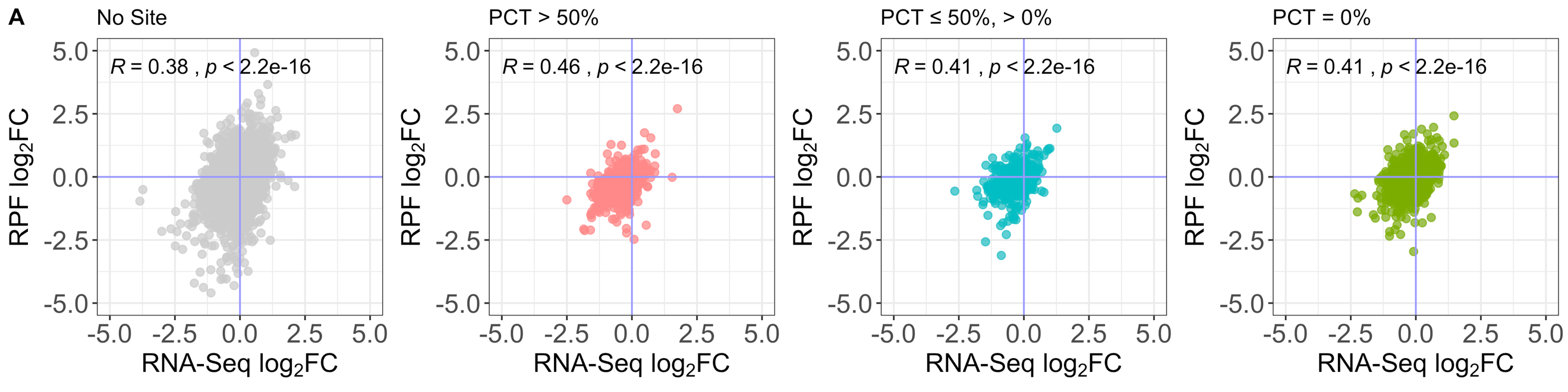

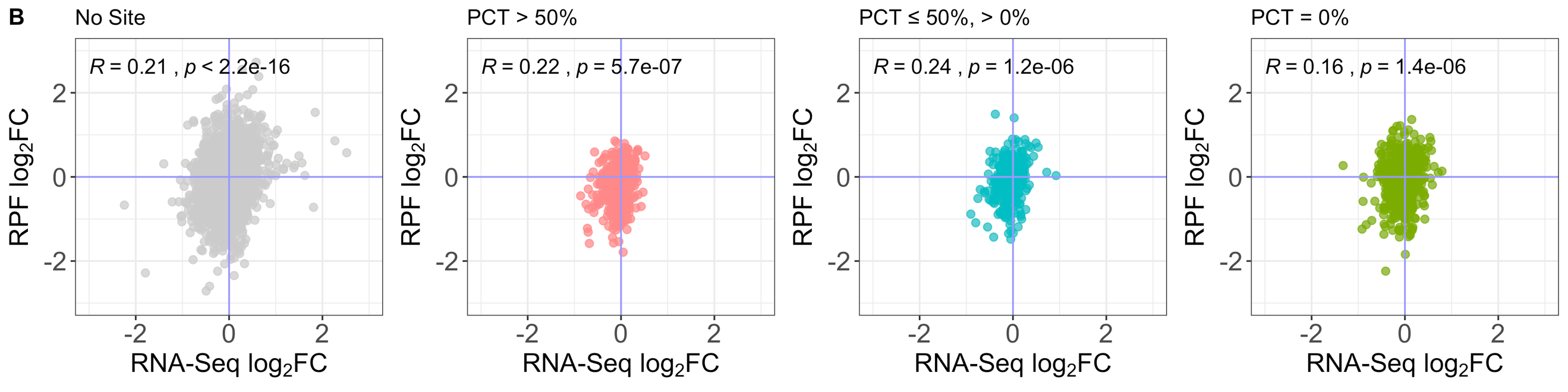

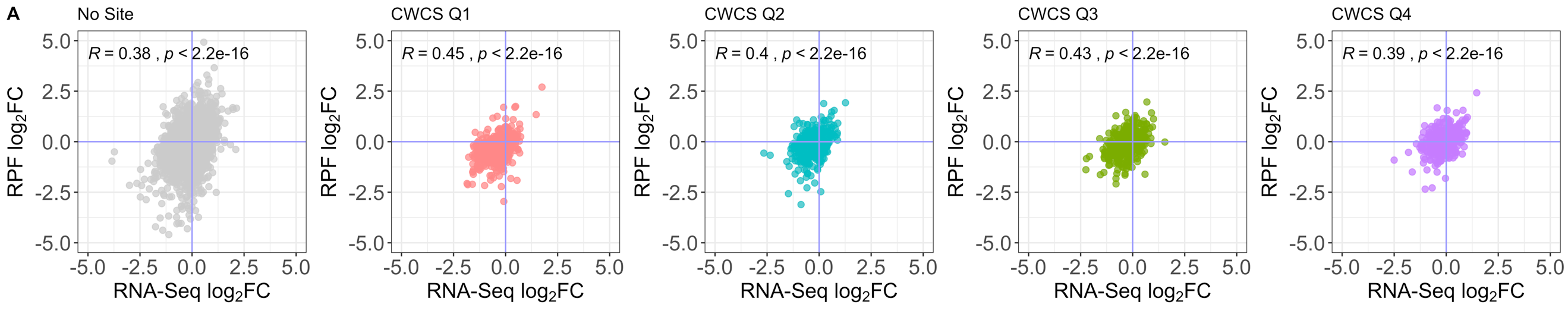

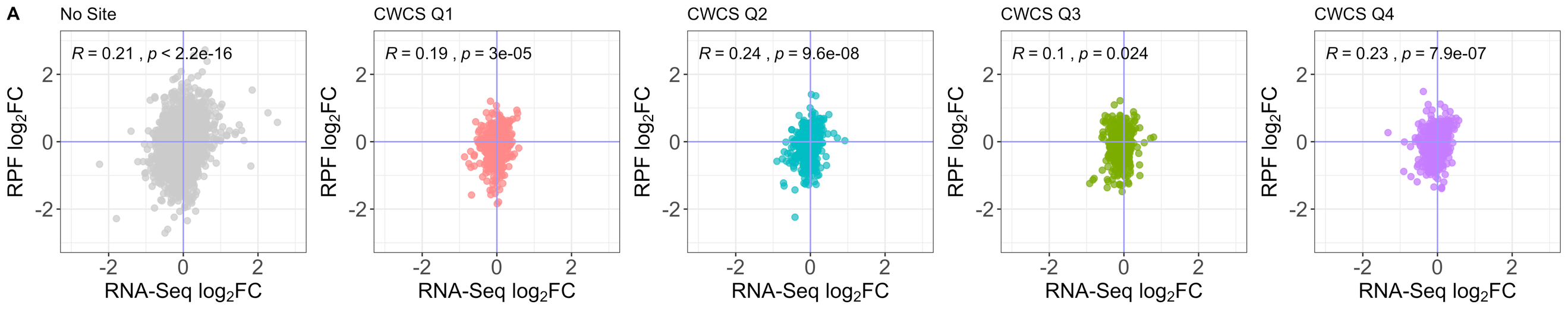


**A**

**B**

**C**

**D**

**Figure S3. Relationship between miR-1271-5p target mRNA abundance and translation. (A & B)** Comparison of miR-1271-5p target gene mRNA and RPF differential expression for the overexpression **(A)** and knockdown **(B)** experiments, after gene stratification via P_ct_ score. Pearson’s correlation coefficients and associated p values are reported top left. **(C & D)** As in **(A & B)**, except after binning target genes into quartiles via cumulative weighted context score.


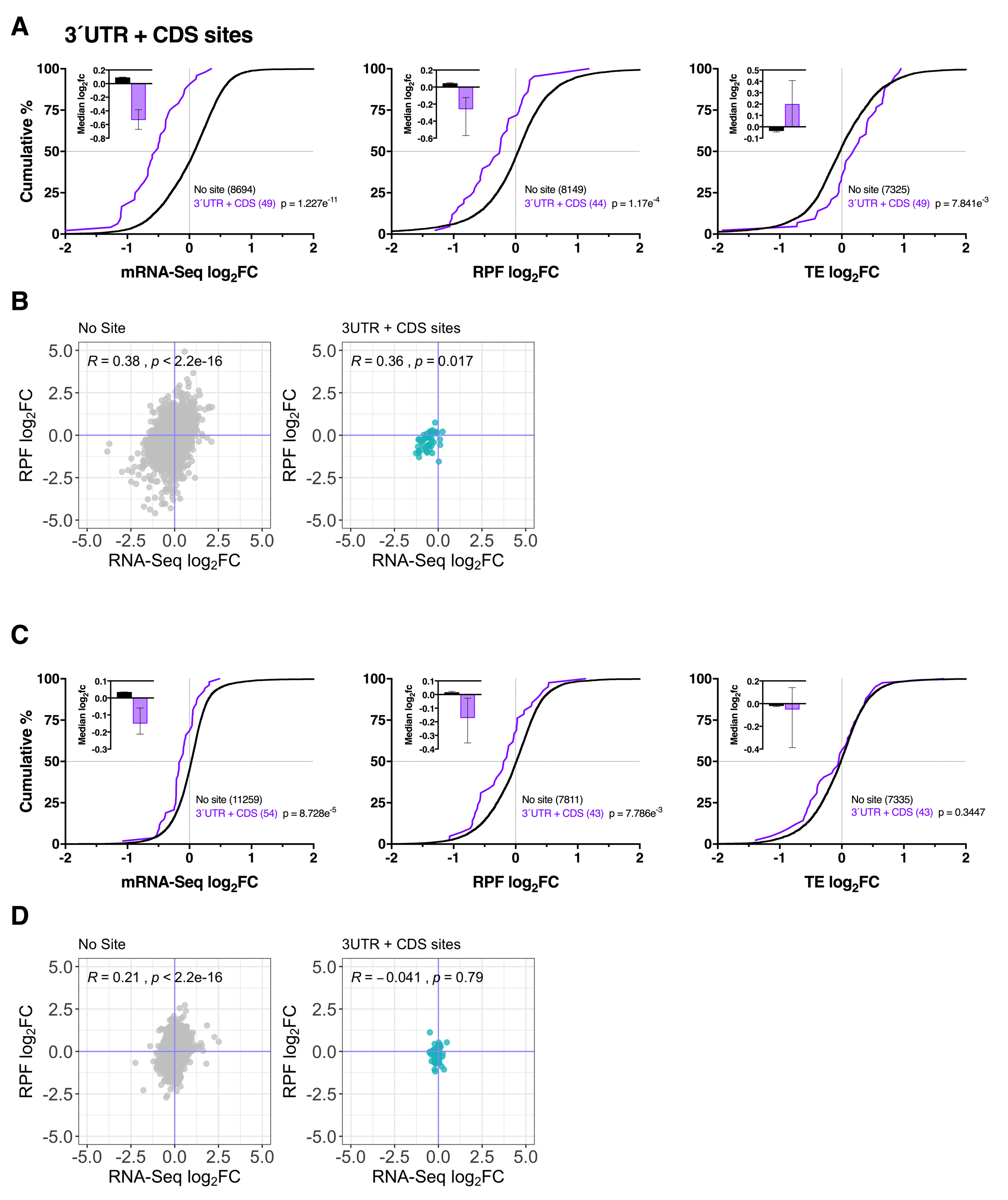


**Figure S4. Combined effect of 3´UTR and CDS miR-1271-5p binding sites on target mRNA expression. (A)** Fold change distributions of target genes containing 3´UTR and CDS binding sites after miR-1271-5p overexpression. To limit the inclusion of low-confidence interactions, only sites with CWCS or TCS < –0.2 were considered. P values calculated relative to genes with no site via two-sided Kolmogorov-Smirnov test. Median log_2_ fold changes ± 95% CI reported top left. **(B)** Comparison of mRNA and RPF changes for genes analysed in **(A).** Pearson’s correlation coefficients and associated p values are reported top left. **(C & D)** As in **(A & B**), except after miR-1271-5p knockdown.

**A**

**B**

**Figure S5. miR-1271-5p target gene interaction networks.** Interaction networks involving miR-1271-5p and target genes significantly downregulated at the mRNA **(A)** or RPF **(B)** level after miR-1271-5p overexpression. Medium confidence molecular interactions amongst target genes were obtained from the STRING database (v11.0) [1] and visualised in Cytoscape (v3.5.1) [2]. Top 50 **(A)** and 44 **(B)** nodes ranked by Edge Percolated Component (EPC) score are shown, with red nodes possessing the highest EPC scores and yellow nodes possessing comparatively lower EPC scores. Red edges represent miRNA-mRNA interactions, whereas black edges correspond to interactions amongst target genes.


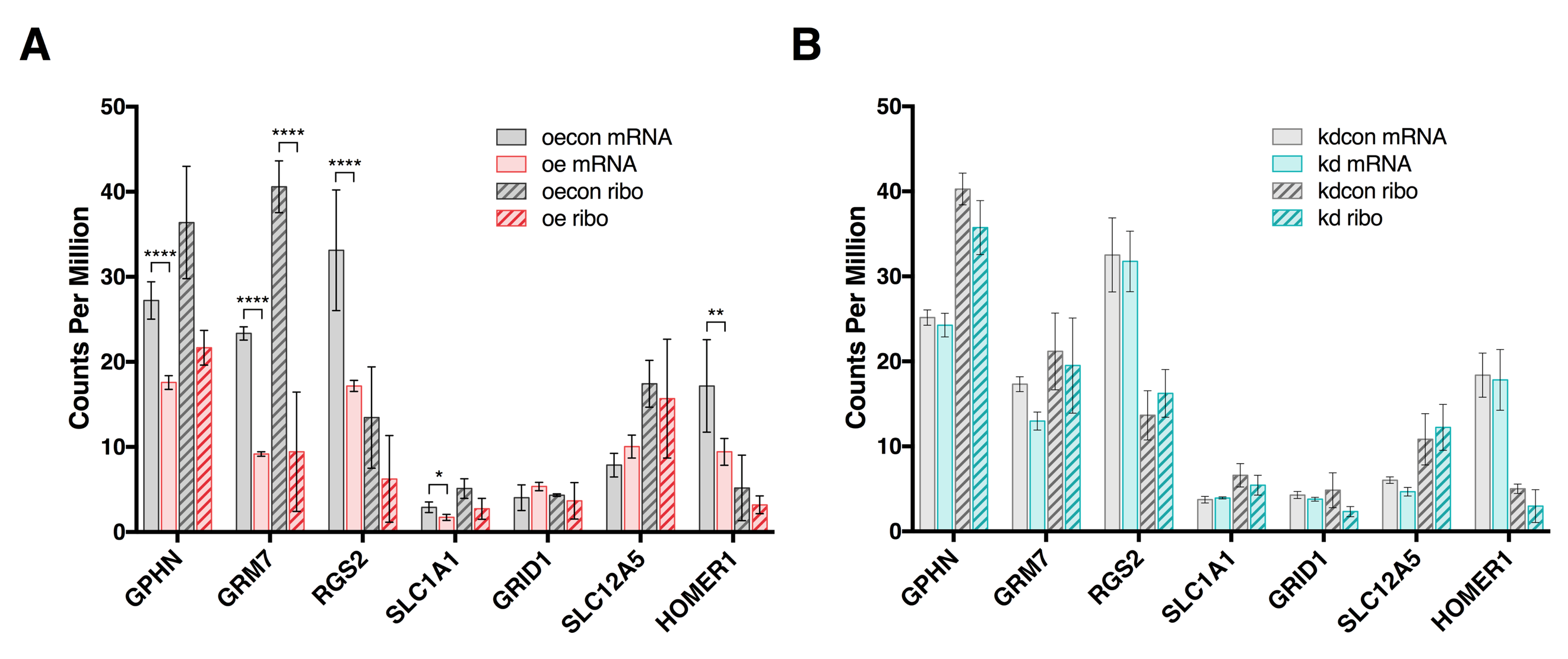


**Figure S6. Differential expression of previously identified miR-1271-5p target genes.** Expression of 7 genes common to our sequencing data sets and a previous low-throughput study conducted by Jensen and Covault [3]. Expression data from the **(A)** overexpression and **(B)** knockdown conditions are shown. Data are presented as counts per million ± SD. mRNA and RPF data were analysed separately via exact test, with FDR < 0.05 and log_2_fc > |±0.5| considered significant (* = FDR < 0.05, ** = FDR < 0.01, **** = FDR < 0.0001).

**REFERENCES**

1. Szklarczyk, D., et al., *STRING v11: protein-protein association networks with increased coverage, supporting functional discovery in genome-wide experimental datasets.* Nucleic Acids Res, 2019. **47**(D1): p. D607-D613.

2. Shannon, P., et al., *Cytoscape: a software environment for integrated models of biomolecular interaction networks.* Genome Res, 2003. **13**(11): p. 2498-504.

3. Jensen, K.P. and J. Covault, *Human miR-1271 is a miR-96 paralog with distinct non-conserved brain expression pattern.* Nucleic Acids Res, 2011. **39**(2): p. 701-11.
