# Supplementary material for: Characterising the Transcriptional and Translational Impact of the Schizophrenia-Associated miR-1271-5p in Neuronal Cells
Source: Cells. 2020 Apr 18;9(4):1014. doi: 10.3390/cells9041014 (PMC7226585; doi:10.3390/cells9041014)
Supplement: Supplementary file 1 [file cells-09-01014-s001.zip › cells-761301.revised.manuscript.supp.material/mir.1271.supp.methods.docx]

**Characterising the Transcriptional and Translational Impact of the Schizophrenia-associated mir-1271-5p in Neuronal Cells**

**Supplementary Methods**

**Ribosome profiling**

Ribosome profiling was conducted using the Illumina TruSeq Ribo Profile kit (H/M/R) kit as per the manufacturer’s instructions, with minor amendments. After miR-1271-5p transfections, cells were incubated in warm culture medium supplemented with 0.1mg/mL cycloheximide (CHX) for 1 min to inhibit translation. Cells were then washed with ice-cold PBS supplemented with CHX, after which 1mL of Mammalian Lysis Buffer (Table 1) was added to each well.

**Table 1. Mammalian Lysis Buffer Formula**

| **Reagent** | **Volume (µL)** |
| --- | --- |
| 5x Mammalian Polysome Buffer | 200 |
| 10% Triton X-100 | 100 |
| 100mM DTT | 10 |
| DNase I (1U/µL) | 10 |
| 50mg/mL cycloheximide | 2 |
| 10% NP-40 | 10 |
| Nuclease-free water | 668 |
| Final volume | 1,000 |

Harvest of cells was then executed via extensive scraping on ice, with lysate collected in fresh tubes chilled on ice, shredded by 25-gauge needle, then incubated on ice for 10 min with periodic inversions. Lysates were subsequently clarified by centrifugation (20,000 x g, 4°C, 10 min) and aliquoted into fresh tubes for ribosome profiling or conventional RNA extractions. At total of ~1mL lysate was recovered from each sample.

For ribosome footprinting, 90U TruSeq Ribo Profile Nuclease (determined by RNase titration) was added to 300µL clarified lysate for 45 min at room temperature with gentle mixing, with reactions stopped via addition of 15µL SUPERase In RNase Inhibitor (Invitrogen). Samples were then concentrated to ~100µL via vacuum centrifugation, after which ribosome protected RNA (RPFs) was purified with MicroSpin S-400 HR columns (GE Healthcare). Columns were first equilibrated with 3mL of 1X Mammalian Polysome Buffer, prior to the addition of 100µL nuclease-digested RPF samples and centrifugation (600 x g, room temp, 2 min). The flow-through was then supplemented with 10µL 10% SDS, following which RNA was purified using a RNA Clean & Concentrator-25 kit (Zymo Research), with samples eluted in 26µL nuclease-free water.

Library preparation was then conducted as per the manufacturer’s instructions. Ribosomal RNA was firstly depleted from purified RPF RNA using the Ribo-Zero rRNA Removal kit (Illumina), with minor amendments to the manufacturer’s protocol. Specifically, the 50°C incubation step during rRNA removal was omitted, while in addition, rRNA-depleted samples were purified via RNA Clean & Concentrator-5 kit (Zymo Research) and eluted in 11µL nuclease-free water. RPFs were then size selected via 12% denaturing urea-polyacrylamide gel electrophoresis (PAGE) as follows. RPF samples were combined with an equal volume of denaturing gel loading dye and denatured at 95C for 5 min, followed by incubation on ice prior to loading. After pre-running the gel for ~20–30 min, samples were loaded and run at 38W for 90 min to ensure defined band separation. Gels were then stained in darkness with SYBR Gold for 5 min at 4°C, briefly visualised under UV transilluminator and bands ~28–30nt were excised. For purification of RPFs, gel slices were shredded and supplemented with nuclease-free water (400µL), 5M ammonium acetate (40µL) and 10% SDS (2µL). After incubation overnight at 4°C with gentle mixing, gel pieces were removed via 0.22µm Costar Spin-X filter tubes (Corning; 2,000 x g, 3 min) and RPFs were precipitated via the addition of glycogen (2µL) and 100% isopropanol (700µL). After incubation at –20°C for 2 hours, samples were centrifuged (12,000 x g, 4°C, 20 min), RNA pellets were washed with fresh, ice-cold 80% EtOH, then air-dried. Samples were subsequently resuspended in 20µL nuclease-free water.

To prepare RPFs for reverse transcription, end repair was conducted by firstly incubating samples with 7.5µL TruSeq Ribo Profile PNK (Polynucleotide Kinase) buffer (94°C, 25 min), followed by the addition of 3µL TruSeq Ribo Profile PNK and 44.5µL nuclease free water, with samples incubated at 37°C for 1 hour. After clean-up via RNA Clean & Concentrator-5 kit, 1µL TruSeq Ribo Profile 3´ adapter was added and samples were heat denatured (65°C, 2 min). Adapter ligation was then performed by supplementing samples with 6µL ligation master mix (3.5µL TruSeq Ribo Profile Ligation Buffer, 1µL 100mM DTT, 1.5µL TruSeq Ribo Profile Ligase) and incubation at 23°C for 2 hours, followed by the addition of 2µL TruSeq Ribo Profile AR Enzyme and further incubation at 30°C for 2 hours.

Reverse transcription was subsequently performed via the addition of 13µL reverse transcription master mix (Table 2), with samples incubated at 50°C for 30 min. 1µL of TruSeq Ribo Profile Exonuclease was then added to each reaction, after which samples were then incubated with the following program: 37°C for 30 min, 80°C for 15 min, 4°C hold. Samples were then diluted to 50µL total volume with nuclease-free water, after which reactions were cleaned-up via RNA Clean & Concentrator-5 kit. Size selection of cDNA fragments was then performed via 10% native page, with 70-100nt fragments purified as above.

**Table 2. Reverse transcription master mix components**

| **Reagent** | **Volume (µL)** |
| --- | --- |
| TruSeq Ribo Profile RT Reaction Mix | 4.5 |
| 100mM DTT | 1.5 |
| Nuclease-free water | 6 |
| EpiScript RT | 1 |
| Final volume | 13 |

Amplification of cDNA libraries prior to sequencing was achieved utilizing a circularisation PCR method to reduce skewing of libraries towards highly abundant cDNAs, which may result in decreased library complexity. cDNA templates were firstly circularised via the addition of CircLigase master mix (Table 3) and incubation at 60°C for 2 hours. A 5µL aliquot of each circularised sample was then combined with PCR master mix (Table 4), with each sample receiving a unique index PCR primer for downstream library multiplexing. A total of 12 PCR cycles were run as per the following program;

98°C for 30 seconds.

12 cycles of:

94°C for 15 seconds.

55°C for 5 seconds.

65°C for 10 seconds.

Final hold at 4°C.

PCR products were then purified using 90µL Agencourt AMPure XP beads (Beckman Coulter) according to the manufacturer’s instructions, after which ~140 – 160bp cDNA libraries were separated from adapter diamers (~113bp) via 8% native PAGE (38W, 70 min, 1% TBE). Libraries were then validated and quantified by running 1µL of each sample on an Agilent High Sensitivity DNA bioanalyzer chip, after which libraries were normalized to 1nM, pooled (10µL of each library). Prior to sequencing, the combined library was denatured with Sodium hydroxide (200mM) and further diluted with HT1-buffer to a final concentration of 1.8pM. This diluted library was subjected to 76 single end sequencing cycles using the Illumina NextSeq500 benchtop sequencer.

**Table 3. cDNA master mix components**

| **Reagent** | **Volume (µL)** |
| --- | --- |
| TruSeq Ribo ProfileCL Reaction Mix | 4 |
| ATP | 2 |
| MnCl_2_ | 2 |
| CircLigase | 2 |
| Final volume | 10 |

**Table 4. PCR master mix components**

| **Reagent** | **Volume (µL)** |
| --- | --- |
| Nuclease-free water | 16 |
| TruSeq Ribo Profile Forward PCR Primer | 2 |
| TruSeq Ribo Profile Index PCR Primer | 2 |
| 2x Phusion Master Mix (NEB) | 25 |
| Final volume | 45 |

**mRNA sequencing**

mRNA sequencing was conducted using the TruSeq Stranded mRNA Library Preparation Kit, according to the manufacturer’s instructions. For each sample, 1µg of high quality (RIN ≥ 8.5) total RNA was diluted to a final volume of 50µL in nuclease-free water and combined with 50µL of oligo-dT RNA purification beads. After gentle mixing, poly(A) RNA was bound to beads via incubation at 65°C for 5 min then room temperature for 5 min. Beads were then concentrated via magnetic stand (5 min), following which supernatant was discarded and unbound RNAs were removed by washing beads with 200µL Bead Washing Buffer (BWB). RNAs bound to oligo-dT beads were then eluted via the addition of 50µL Elution Buffer and incubation at 80°C for 2 min, followed by a 25°C hold. To further purify mRNA from non-specifically bound rRNAs, mRNA was rebound to oligo-dT beads with 50µL Bead Binding Buffer, samples were incubated (room temperature, 5 min), beads were pelleted via magnetic stand and unbound RNA was removed via washing with 200µL BWB.

To fragment and prime the remaining mRNA for reverse transcription, beads were resuspended in 19.5µL Fragment, Prime, Finish Mix and incubated at 94°C for 8 min. Magnetic beads were then pelleted and 17µL of supernatant was collected from each sample for further processing. For first strand synthesis, 8µL of First Strand Synthesis Act D Mix with SuperScript II (1µL SuperScript II per 9µL FSS Act D Mix) was added to each sample and reactions were developed under the following conditions; 25°C for 10 min, 42°C for 15 min, 70°C for 15 min, 4°C hold. Second strand synthesis was immediately conducted by adding 20µL of Second Strand Marking Master Mix followed by incubation at 16°C for 1 hour. Samples were then brought up to room temperature and cDNA was purified using 90µL AMPure XP beads (Beckman Coulter), according to the manufacturer’s instructions, with cDNA eluded in 17.5µL Resuspension Buffer (~15µL recovered per sample).

Single adenosine nucleotides were then added to 3´ ends for prior to adapter ligation by adding 12.5µL A-Tailing Mix and incubating samples as per the following program; 37°C for 30 min, 70°C for 5 min, 4°C hold. Adapter ligation was immediately performed by adding 2.5µL Ligation Mix and 2.5µL RNA Adapter Index, with each sample receiving a unique adapter index sequence for downstream library demultiplexing. Reactions were then incubated at 30°C for 10 min and halted via the addition of 5µL Stop Ligation Buffer. Adapter ligated cDNA was then purified via using 42µL AMPure XP Beads, with samples eluted in 52.5µL Resuspension Buffer (~50µL recovered per sample). To ensure successful purification of cDNA, a second purification step was performed using 50µL AMPure XP Beads, with samples eluted in 22.5µL Resuspension Buffer (~20µL recovered per sample).

PCR amplification was next conducted by supplementing samples with 5µL PCR Primer Cocktail and 25µL PCR Master Mix, followed by 15 cycles of PCR as per the following program;

98°C for 10 seconds.

15 cycles of;

98°C for 10 seconds.

60°C for 30 seconds.

72°C for 30 seconds.

72°C for 5 min.

Hold at 4°C.

Amplified cDNA was subsequently purified using 50µL AMPure XP Beads, with samples eluted in 32.5µL Resuspension Buffer (~30µL recovered per sample). Libraries were then validated and quantified by running 1µL of each sample on an Agilent High Sensitivity DNA bioanalyzer chip, after which libraries were normalized to 2nM, pooled (10µL of each library). Prior to sequencing, the combined library was denatured with Sodium hydroxide (200mM) and further diluted with HT1-buffer to a final concentration of 1.8pM. This diluted library was subjected to 151 single end sequencing cycles using the Illumina NextSeq500 benchtop sequencer.

**Processing of sequencing data**

*Ribosome profiling*

Raw bcl files obtained via the NextSeq 500 were in Phred33 encoding, stranded and single end. These files were demultiplexed and converted to fastq format via *Bcl2fastq* (version 2.2, Illumina) using the following command;

bcl2fastq --sample-sheet <sample.sheet>.csv -o <output.directory>

Note that no adapter sequences were included in the sample sheet to prevent automatic adapter trimming, which masks adapter sequences if the final read is < 35 nt. Output fastq files were subsequently decompressed and lane data were then merged for each sample. Quality control reports were then generated via *FastQC* (version 0.11.5) with the following command;

fastqc <input>.fastq

The *FastQC* reports were manually inspected for per base quality, as well as overrepresented sequences and adapter content. Removal of adapters was next executed via *Cutadapt* (version 1.14);

cutadapt -A AGATCGGAAGAGCACACGTCT -o <output>.fastq <input>.fastq

After assessing efficacy of adapter removal by *FastQC*, *Cutadapt* was then used to further process fastq files. Although read quality was exceptionally high for all samples, reads were trimmed from the 3´ end based on Phred33 quality score to ensure all 3´ ends were of good quality. In addition, reads between 25 – 40nt were selected for further analysis;

cutadapt --nextseq-trim 20 -m 25 -M 40 -o <output>.fastq <input>.fastq

Since single 5´ nucleotides are often added in an untemplated manner during library preparation [1], these were next removed by *Cutadapt*;

cutadapt -u 1 -o <output>.fastq <input>.fastq

As Ribo-Seq libraries often exhibit unintended carry-over of small non-coding RNAs, we next aligned processed reads to a fasta reference file (containing rRNA, miRNA, snRNA and snoRNA sequences) with *Bowtie2* (version 2.2.6) and retained unaligned reads for further analysis;

bowtie2 -L 20 --un <unaligned.reads>.fastq -x </path/to/noncoding.RNA.bt2.index> -U <input>.fastq -S <noncoding.RNA.alignments>.sam

Alignment to the reference genome (NCBI GRCh38, obtained from Illumina iGenomes) was then conducted using *Tophat2* (version 2.2.1), with the output encoded in bam format (default);

tophat --GTF <path/to/ncbi.grch.38.gtf --num-threads 1 --no-novel-juncs --output-dir <output.directory> <input>.fastq

Finally, reads aligning to features were counted using *HTSeq* (version 0.7.2). Using default parameters, reads were assumed to be stranded and sense relative to the genome (as indicated by Illumina). To ensure that small noncoding RNA reads *not* removed by *Bowtie2* were not counted, a reference genome with rRNA, miRNA, snRNA and snoRNA loci omitted was used to guide read-counting;

htseq-count -f bam <input>.bam <reference.genome>.gtf > <read.counts>.txt

*mRNA sequencing*

Demultiplexing of single stranded mRNA libraries was conducted with *Bcl2fastq*, with adapter sequences specified to allow for automatic adapter trimming. Merging, QC and 3´ end trimming were then performed as above. For genome alignment, *HISAT2* (version 2.1.0) and *samtools* (version 1.9) were used as follows;

hisat2 --dta -x <path/to/ncbi/grch38/hisat2/index> -U <input>.fastq -S <output>.sam

samtools view -bS <output.sam> | samtools sort - <output.sorted>.bam

Read-counting was next undertaken with *HTSeq*, with strandedness set to ‘reverse’ as these reads are antisense to the genome;

htseq-count -s “reverse” -f <input.file.format> <input>.bam <reference.genome>.gtf > <read.counts>.txt

*Analysis of alignment read distribution*

The number of reads aligning to genomic features (i.e. transcription start/stop sites, 5´UTR, CDS, 3´UTR) was conducted with RSeQC (version 2.6.4) as follows;

read_distribution.py -i <input>.bam -r hg38_Refseq.bed >> <output>.txt

*Metagene analysis and subcodon phasing*

For analysis of metagene alignment, regions of interest were firstly generated around start (50bp upstream, 300bp downstream) and stop (300bp upstream, 50bp downstream) using the *metagene generate* subscript of the *Plastid* python library;

metagene generate --annotation_files grch38.gtf --landmark cds_<start/stop> --upstream <50/300> --downstream <300/50> <roi.name>

Reads aligning around these features were then counted with *metagene count*. For this analysis, P-site offset was defined as 14nt from the 5´ end;

metagene count <start/stop.roi>.txt <output.name> --count_files <input>.bam --fiveprime --offset 14 --normalize_over <-50-300/-300-50> --min_count 50 --use_mean --max_length 151 (mRNA only)

For subcodon phasing, reads were phased around start codons using the script *phase_by_size.py* as follows;

phase_by_size.py cds.start.50.300.roi.txt <output.name> --count_files <input>.bam --fiveprime --offset 14

**Differential expression analysis**

Raw count files (*HTSeq*) from each sample were merged into a single read-count matrix, and reads classed as “no_feature”, “ambiguous”, “too_low_aQual”, “not_aligned” or “alignment_not_unique” were removed. Differential mRNA or RPF expression was then calculated via *EdgeR* (version 3.28.0) as follows. The merged read-count matrix was firstly imported into R and converted into an *EdgeR* DGEList object. A minimum counts-per-million (CPM) threshold was then used to filter out genes with low read counts across all samples. This threshold ensured that genes with a raw read count of 5 in the smallest library were removed. Library normalization factors were then calculated via trimmed mean of means (TMM) method and dispersion was estimated, after which differential expression was determined via exact test. The general analysis pipeline is presented below;

# Import edgeR library

library("edgeR")

# Import merged counts

x <- read.delim("merged.counts.txt", row.names="gene_id")

# Assign groups, matching order of columns in merged count file

group <- c("C","C","C","K","K","K")

# Create DGEList object

y <- DGEList(counts=x,group=group)

# Filter out genes with low read-counts across all samples

keep <- rowSums(cpm(y)>1.5) >= 3

y2 <- y[keep,,keep.lib.sizes=FALSE]

# Calculate normalization factors and estimate dispersion

y3 <-calcNormFactors(y2)

y3 <- estimateDisp(y3)

# Determine differential expression via exact test

et <- exactTest(y3, c("C", "K"))

Analysis of differential translational efficiency was conducted via the *RiboDiff* (version 0.2.2) package. Raw mRNA and RPF counts were firstly merged into a single matrix, after which genes surviving *edgeR* CPM thresholds were selected for further analysis. A csv file containing sample names, data types (mRNA-Seq/Ribo-Seq) and experimental conditions (overexpression control/overexpression or knockdown control/knockdown) was additionally produced to guide translational efficiency analysis. Translational efficiency was then calculated using the following script, with a p value threshold set to 1 to ensure all results were printed;

python ribodiff/scripts/TE.py -p 1 -e <phenotype.file>.csv -c <merged.counts>.txt -o <output>.txt

**Identification of mRNAs with CDS miR-1271-5p binding sites**

mRNAs containing miR-1271-5p binding sites in the CDS were predicted using perl custom prediction scripts from the TargetScan (version 6) database. This version was favoured over newer versions, which heavily assume query mRNA sequences are derived from the 3´UTR. Note that P_ct_ scores were not calculated, as the provided script uses pre-calculated 3´UTR conservation data to guide P_ct_ scoring.

Predicted binding sites were firstly generated using the *targetscan_60.pl* script, with text files containing all human representative transcript ORF sequences (TargetScan) and the miR-1271-5p seed region used as input;

./targetscan_60.pl mir.1271.family.info.txt hsa.rep.trans.orf.seq.txt <output>.txt

Binding site context+ scores were then calculated using the *targetscan_60_context_scores.pl* script, with text files containing the full miR-1271-5p sequence, ORF sequences and targetscan_60.pl output used as input;

./targetscan_60_context_scores.pl mir.1271.seq.txt hsa.rep.trans.orf.seq.txt targetscan_60.output.txt <output>.txt

miR-1271-5p-mRNA interactions were then printed to a text file containing gene ids and context+ scores in tab delimited format. This file was then imported into R and context+ scores for each gene were added to produce total context+ scores, as previously described;

# Import context+ scores

x<-read.delim("context.scores.txt", stringsAsFactors = FALSE)

y<-transform(x, context.score = as.numeric(context.score))

# Convert scores > 0 to 0 (as per Garcia et al)

y$context.score[y$context.score > 0] <- 0

# Sum context+ scores per gene

z<-aggregate(context.score~Gene.ID, data=y, FUN=sum)

**References**

1. Ingolia, N.T., et al., *The ribosome profiling strategy for monitoring translation in vivo by deep sequencing of ribosome-protected mRNA fragments.* Nat Protoc, 2012. **7**(8): p. 1534-50.
2. Garcia, D.M., et al., *Weak seed-pairing stability and high target-site abundance decrease the proficiency of lsy-6 and other microRNAs.* Nat Struct Mol Biol, 2011. **18**(10): p. 1139-46.
